# Brain age in genetic and idiopathic Parkinson's disease

**DOI:** 10.1093/braincomms/fcae382

**Published:** 2024-12-20

**Authors:** Stefan J Teipel, Hauke Hoffmann, Alexander Storch, Andreas Hermann, Martin Dyrba, Julia Schumacher

**Affiliations:** Deutsches Zentrum für Neurodegenerative Erkrankungen (DZNE) Rostock-Greifswald, Rostock 18147, Germany; Department of Psychosomatic Medicine, University Medical Center Rostock, Rostock 18147, Germany; Department of Psychosomatic Medicine, University Medical Center Rostock, Rostock 18147, Germany; Deutsches Zentrum für Neurodegenerative Erkrankungen (DZNE) Rostock-Greifswald, Rostock 18147, Germany; Department of Neurology, University Medical Center Rostock, Rostock 18147, Germany; Deutsches Zentrum für Neurodegenerative Erkrankungen (DZNE) Rostock-Greifswald, Rostock 18147, Germany; Department of Neurology, University Medical Center Rostock, Rostock 18147, Germany; Translational Neurodegeneration Section ‘Albrecht Kossel’, Department of Neurology, University Medical Center Rostock, Rostock 18147, Germany; Deutsches Zentrum für Neurodegenerative Erkrankungen (DZNE) Rostock-Greifswald, Rostock 18147, Germany; Deutsches Zentrum für Neurodegenerative Erkrankungen (DZNE) Rostock-Greifswald, Rostock 18147, Germany; Department of Neurology, University Medical Center Rostock, Rostock 18147, Germany

**Keywords:** glucocerebrosidase gene (GBA) mutation, leucine-rich repeat kinase 2 gene (LRRK2) mutation, brain volume, MRI, atrophy

## Abstract

The brain-age gap, i.e. the difference between the brain age estimated from structural MRI data and the chronological age of an individual, has been proposed as a summary measure of brain integrity in neurodegenerative diseases. Here, we aimed to determine the brain-age gap in genetic and idiopathic Parkinson's disease and its association with surrogate markers of Alzheimer's disease and Parkinson's disease pathology and with rates of cognitive and motor function decline. We studied 1200 cases from the Parkinson's Progression Markers Initiative cohort, including idiopathic Parkinson's disease, asymptomatic and clinical mutation carriers in the leucine-rich repeat kinase 2 gene (LRRK2) and the glucocerebrosidase gene (GBA), and normal controls using a cohort study design. For comparison, we studied 187 Alzheimer's disease dementia cases and 254 controls from the Alzheimer's Disease Neuroimaging Initiative cohort. We used Bayesian ANOVA to determine associations of the brain-age gap with diagnosis, and baseline measures of motor and cognitive function, dopamine transporter activity and CSF markers of Alzheimer's disease type amyloid-β42 and phosphotau pathology. Associations of brain-age gap with rates of cognitive and motor function decline were determined using Bayesian generalized mixed effect models. The brain-age gap in idiopathic Parkinson's disease patients was 0.7 years compared to controls, but 5.9 years in Alzheimer's disease dementia cases. In contrast, asymptomatic LRRK2 individuals had a 1.1. year younger brain age than controls. Across all cases, the brain-age gap was associated with motor impairment and (in the clinically manifest PD cases) reduced dopamine transporter activity, but less with CSF amyloid-β42 and phosphotau. In idiopathic Parkinson's disease cases, however, the brain-age gap was associated with lower CSF amyloid-β42 levels. In sporadic and genetic Parkinson's disease cases, a higher brain-age gap was associated with faster decline in episodic memory, and executive and motor function, whereas in asymptomatic LRRK2 cases, a smaller brain-age gap was associated with faster cognitive decline. In conclusion, brain age was sensitive to Alzheimer's disease like rather than Parkinson's disease like brain atrophy. Once an individual had idiopathic Parkinson's disease, their brain age was associated with markers of Alzheimer's disease rather than Parkinson's disease. Asymptomatic LRRK2 cases had seemingly younger brains than controls, and in these cases, younger brain age was associated with poorer cognitive outcome. This suggests that the term brain age is misleading when applied to disease stages where reactive brain changes with apparent volume increases rather than atrophy may drive the calculation of the brain age.

## Teaser

Teipel *et al*. report increased brain age in Parkinson's disease, associated with cognitive decline. Surprisingly, asymptomatic carriers of a Parkinson's risk gene showed younger brains. These findings suggest accelerated brain aging in Parkinson's disease, but indicate a knowledge gap in understanding brain age in cases of genetic risk.

## Graphical Abstract

**Graphical Abstract:**
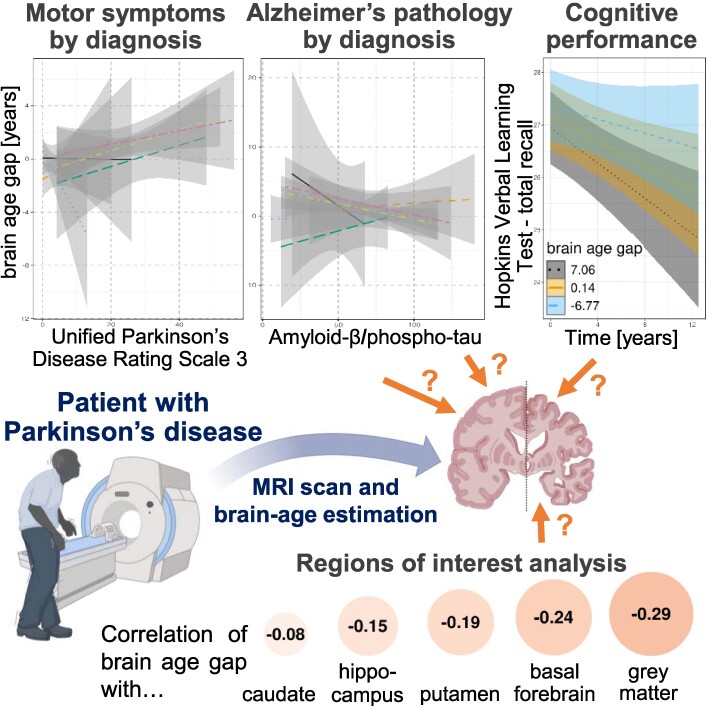

## Introduction

Parkinson's disease (PD) is the second most common neurodegenerative disease in humans after Alzheimer's disease (AD).^1^ In a subset of cases, PD is associated with cognitive decline and dementia, with a prevalence of 24–31% of PD cases presenting with dementia.^2^ AD co-pathology was estimated to be present in 10% of PD, 30% of PD dementia and 70% of Lewy body dementia cases,^3^ and AD pathology was found to contribute to cognitive decline in PD.^4^

The brain-age gap is the difference between the brain age estimated from structural MRI data and the chronological age of an individual. It has been proposed as a summary measure of brain integrity in neurodegenerative diseases.^5^ A positive brain-age gap indicates that a person's brain structure is older than their chronological age, a negative brain-age gap indicates that the brain is younger than its chronological age. Brain age has been widely studied in neurodegenerative diseases such as AD^6-8^ and Amyotrophic Lateral Sclerosis,^9^ as well as in depression and schizophrenia.^10^ It was found to be associated with trajectories of cognitive decline^7,11^ and molecular markers of pathology in AD.^8,12^ Two previous studies in subsamples of the Parkinson's Progression Markers Initiative (PPMI) cohort^13^ reported an increased brain-age gap of 2.9 years and 1.5 years, respectively, in idiopathic PD cases that was associated with cognitive and motor impairment and with disease duration.^14,15^ In a small study, the brain-age gap in 33 PD patients was +0.75 years.^16^ In a study on the LANDSCAPE cohort,^17^ the brain-age gap was +2.2. years in PD cases without and +3.5 years in PD cases with dementia.^18^ The brain-age gap in this study^18^ was associated with longitudinal decline of attention and working memory performance. Supplementary Table 1 provides an overview of previous studies on brain age in PD.

PD is increasingly recognized as a genetic disease with more than 25 genetic variants that have been shown to be causal for PD, and more than 20 genetic risk loci identified in PD genome wide association studies.^19^ Mutations of the glucocerebrosidase (GBA) gene account for 5–15% of clinical PD cases, rendering it numerically the most important genetic risk factor for PD.^20^ These mutations can lead to a loss of glucocerebrosidase activity and lysosomal dysfunction.^20^ More than 300 pathogenic GBA mutations have been identified that were found to be associated with different pathogenetic pathways that lead to downstream accumulation of alpha-synuclein.^21^ Clinically, GBA mutations are associated with early-onset and rapidly progressing PD that phenotypically resembles idiopathic PD but with higher frequency of cognitive decline.^22-25^ Almost all GBA mutation carriers have Lewy body pathology,^26^ and GBA mutations are a significant risk factor for dementia with Lewy bodies, where they are associated with a more pure alpha-synucleinopathy and less AD co-pathology.^27,28^

Mutations in the leucine-rich repeat kinase 2 (LRRK2) gene lead to a toxic gain of function of the LRRK2 kinase and influence the accumulation of alpha-synuclein.^29^ LRRK2 mutations have been associated with late-onset PD, resembling idiopathic PD with a predominant motor phenotype.^30^ In PD with LRRK2 mutations, the prevalence of Lewy body pathology is more variable and related to the presence of cognitive impairment while a primarily motor phenotype can occur in the absence of alpha-synuclein pathology,^26,31^ Both GBA and LRRK2 mutations associated with genetic PD provide a window into prodromal stages when considering clinically asymptomatic mutation carriers.

Here, we extended previous studies on the brain-age gap in PD^14-16,18^ by determining differential contribution of molecular and behaviour markers of PD and AD pathology to the brain-age gap in PD cases, examining the brain-age gap in asymptomatic and clinical PD cases with GBA and LRRK2 mutations and identifying associations of the brain-age gap with domain specific cognitive decline. We expected an increased brain age in PD, but also already in asymptomatic mutation carriers, which would be associated with accelerated cognitive and motor decline. We estimated brain age from MRI data of 1200 cases retrieved from the PPMI cohorts, including controls, asymptomatic and clinically manifest GBA and LRRK2 mutation carriers and idiopathic PD cases. We used James H. Cole's brain age estimation algorithm,^32^ which has previously been validated,^33^ and used an independent sample of AD patients and controls from the ADNI cohort to relate the observed brain-age gap differences in PD. We used a Bayesian analysis framework to directly quantify the evidence for and against an effect.^34^

## Material and methods

### Data sources

The data of PD cases and matched controls came from the Parkinson's Progression Markers Initiative (PPMI) database (www.ppmi-info.org/access-data-specimens/download-data). The PPMI cohorts are described in detail at www.ppmi-info.org. The data of AD cases and matched controls came from the Alzheimer's Disease Neuroimaging Initiative (ADNI) cohorts, accessed via the ADNI database (http://adni.loni.usc.edu).

### Consent statement

For both studies, PPMI and ADNI, written informed consent was obtained from all participants and/or authorized representatives. The study protocols for both studies had been approved by the local institutional review boards and ethical committees of the centres participating in the respective study. PPMI and ADNI are being conducted in accordance with the Helsinki Declaration of 1975 and its later amendments.

### Participants

We retrieved data openly available data from PPMI of 1200 cases with MRI scans at baseline, including 211 healthy controls, 499 cases with idiopathic PD (defined here as PD without mutations in GBA, LRRK2, SNCA, Parkin, or PINK1), 149 asymptomatic and 112 clinically manifest LRKK2 mutation carriers, as well as 169 asymptomatic and 60 clinically manifest GBA mutation carriers (Table 1). Diagnosis of idiopathic Parkinson's disease was based on the UK Parkinson's Disease Society Brain Bank Clinical Diagnostic Criteria^35^ following a thorough diagnostic work-up, including neurological examination, medical history and family anamnesis and neuropsychiatric assessment. In turn, non-idiopathic Parkinsonism was an exclusion criterion. PPMI idiopathic PD patients were unmedicated and not expected to require Parkinson's disease medication within at least 6 months per the study protocol. Asymptomatic genetic cases were known to carry a risk variant of LRRK2 or GBA, but had no clinical diagnosis of PD or other parkinsonism or dementia. At baseline, healthy controls had no current or active clinically significant neurological disorder, no first-degree relative with PD and normal dopamine transporter (DAT) SPECT imaging by visual inspection.

**Table 1:** Demographics of PPMI cases

	*N* (f/m)^a^	Age [years]^b^ mean (SD)	Education [years]^c^ mean (SD)	UPDRS3 off mean (SD)^d^	MoCA mean (SD)^e^	GDS mean (SD)^f^
Controls	211 (77/134)	60.94 (11.40)	16.05 (3.00)	1.14 (2.05)	28.02 (1.45)	1.26 (2.19)
PD idiopathic	499 (176/323)	62.39 (9.74)	16.01 (3.17)	21.47 (10.33)	27.02 (2.41)	2.23 (2.46)
Asymptomatic LRRK2	149 (89/60)	61.19 (6.91)	16.80 (3.71)	2.62 (3.92)	27.07 (2.15)	1.73 (2.32)
LRKK2-PD	112 (51/61)	63.76 (8.98)	15.48 (4.59)	22.01 (10.63)	26.01 (3.06)	3.17 (3.13)
Asymptomatic GBA	169 (103/66)	61.80 (6.67)	16.60 (3.27)	2.62 (3.87)	26.77 (2.22)	1.97 (2.61)
GBA-PD	60 (26/34)	61.47 (10.97)	17.98 (2.58)	27.95 (10.90)	26.48 (2.57)	2.65 (2.87)

^a^Evidence is extremely in favour of a difference of sex distribution across diagnoses, BF_10_ = 1.5 ∗ 10^7^.

^b^Evidence is strongly in favour of no difference of age across diagnoses, BF_10_ = 0.024.

^c^Evidence is extremely in favour of a difference of education across diagnoses, BF_10_ = 1.8 ∗ 10^8^.

^d^Evidence is extremely in favour of a difference of UPDRS-3 off scores across diagnoses, BF_10_ = 3.3 ∗ 10^234^.

^e^Evidence is extremely in favour of a difference of MoCA scores across diagnoses, BF_10_ = 6.7 ∗ 10^9^.

^f^Evidence is extremely in favour of a difference of GDS scores across diagnoses, BF_10_ = 4.5 ∗ 10^6^.

As reference, we retrieved data of 187 AD dementia cases and 254 controls from the ADNI cohort (Table 2). These data were used as a benchmark for the PD findings. We did not aim to include all ADNI cases, but rather a substantial number of AD patients and controls from the ADNI2 and ADNI3 cohorts with good quality MRI scans. ADNI cognitively normal subjects had MMSE scores between 24 and 30 (inclusive), a CDR = 0, were non-depressed, non-MCI, and non-demented, and reported no subjective memory concerns. AD dementia cases had MMSE scores between 20 and 26 (inclusive), a CDR = 0.5 or 1.0 with impaired activities of daily living and fulfilled NINCDS-ADRDA criteria for clinically probable Alzheimer's disease.^36^

**Table 2:** Demographics of ADNI cases

	*N* (f/m)^a^	Age [years]^b^ mean (SD)	Education [years]^c^ mean (SD)	MMSE mean (SD)^d^
Controls	254 (130/124)	75.4 (6.6)	16.4 (2.7)	29.1 (1.2)
AD dementia	187 (79/108)	75.1 (7.8)	15.9 (2.6)	22.6 (3.2)

^a^Evidence is in favour of no difference of sex distribution between diagnoses, BF_10_ = 0.66.

^b^Evidence is in favour of no difference of age between diagnoses, BF_10_ = 0.12.

^c^Evidence is in favour of no difference of education between diagnoses, BF_10_ = 0.51.

^d^Evidence is extremely in favour of a difference of MMSE score between diagnoses, BF_10_ = 1.47 ∗ 10^102^.

Sample size was not based on a priori power calculation, but on availability of data.

### Neuropsychological testing

From the PPMI database, we retrieved scores for neuropsychological tests of episodic memory, as a cognitive marker of an AD pathology component, and attention and working memory as cognitive markers of a PD pathology component. For episodic memory, we used the Hopkins Verbal Learning Test (HVLT) total recall score,^37^ for processing speed and attention, we used the Symbol Digits Modalities Test (SDMT),^38^ for working memory the letter–number sequencing subtest^39^ of the fourth edition of the Wechsler Adult Intelligence Scale (WAIS-IV), for visuospatial perception the Benton Judgment of Line Orientation test^38^ and for executive function the semantic verbal fluency test.^38^ In addition, we used the Montreal Cognitive Assessment (MoCA)^40^ score for global cognition. For assessment of depression, we used data on the Geriatric Depression Scale (GDS). For motor impairment, we retrieved scores of part 3 of the Unified Parkinson's Disease Rating Scale (UPDRS3).

### Striatal dopamine transporter binding acquisition and pre-processing

DaTSCAN ([(123I)-FP-CIT SPECT] imaging was performed in all cases, but is publicly available only for the idiopathic PD, LRRK2-PD and GBA-PD cases and controls, but not for the asymptomatic LRRK2 and GBA cases. Scans were obtained at PPMI imaging centres and sent to the imaging core for processing and calculation of striatal binding ratios (SBRs) for striatal subregions (putamen, caudate nucleus). Details of data acquisition and pre-processing are described in the PPMI SPECT Technical Operations Manual (https://www.ppmi-info.org/sites/default/files/docs/PPMI2.0_SPECT_TOM_Final_v6.0_20221201_FE.pdf) and previous publications.^41,42^

We did not perform the DaTSCAN data processing ourselves, but retrieved the regional striatal binding ratios from the PPMI data repository. To reduce the dimensionality of the data, we performed a principal component analysis of the z-standardized binding ratios of the left and right caudate and putamen, using the ‘prcomp’ command in R. The first principal component accounted for 88% of the variance across the regions and was used as a representative of the SBR in subsequent analyses.

### MRI data

In PPMI and ADNI, structural MRI scans were collected using harmonized imaging protocols. Participating sites acquired T_1_-weighted MP-RAGE or IR-FSPGR sequences using Siemens, GE, or Philips MRI scanners with 1.5 T or 3 T magnetic field strength. Sagittal plane resolution was 1.0 mm × 1.0 mm voxel size and included 192 slices with thickness between 1.0 and 1.3 mm. Other parameters such as repetition and echo time followed the manufacturer's recommendations for a T_1_-weighted, 3D sequence at each site. See https://www.ppmi-info.org/study-design/research-documents-and-sops and https://adni.loni.usc.edu/wp-content/themes/freshnews-dev-v2/documents/mri/ADNI_MRI_overview_2.6.18.pdf for further details about the imaging procedures. All scans were visually inspected to check to exclude data with insufficient image quality or artefacts.

### MRI data processing and brain age calculation

The software ‘brainageR’ (v2.1)^32^ was used to estimate the brain-age scores. We used the algorithm of Cole's brainageR toolbox because (i) we already had experience with it from a previous study in patients with Amyotrophic Lateral Sclerosis (allowing comparison of results);^9^ (ii) the algorithm is among the most widely validated and used; and (iii) it is easy accessible and reproducible through the R library, https://github.com/james-cole/brainageR. First, the automated brainageR pipeline processed the T_1_-weighted images using SPM12 (r7487, Wellcome Centre for Human Neuroimaging, London, UK), including the steps (i) segmentation into grey matter, white matter and CSF compartments; (ii) high-dimensional non-linear DARTEL normalization to the brain template provided by brainageR; and (iii) final smoothing by a 4 mm Gaussian kernel. Then, a principal component transformation was applied to the smoothed images of the grey matter, white matter and CSF. Finally, the transformed data were entered into a pre-trained Gaussian progression regression model to obtain the brain age estimates. The difference between the estimated and chronological age was labelled as the brain-age gap, with a positive value indicating an older-appearing brain and a negative value indicating a younger-appearing brain.

To reduce potential proportional bias of brain age estimates in younger or older participants, we corrected the raw brain-age score using a linear regression model, which was fitted on the control subjects.^6,10^  $\mathrm{brainag}e_{\mathrm{con}}=\alpha +\beta *\mathrm{bioag}e_{\mathrm{con}}$. This procedure was conducted separately for both PPMI and ADNI studies. The model intercept *α* and slope *β* terms were then applied to the whole sample to correct the brain-age scores^43^: corrbrainage=brainage+[bioage−(α+β*bioage)], with the second part in square brackets representing the calibration term (=residual for the control subjects). We checked proper calibration, i.e. that the corrected brain-age scores on average matched the actual biological age of the controls and that the residuals (=brain-age gap values) were evenly distributed across all ages, i.e. homoscedastic.

The difference between the corrected brain age and the chronological age was termed the brain-age gap, with a positive value indicating an older-appearing brain and a negative value indicating a younger-appearing brain. Given the raw brain-age values derived from brainageR, we estimated a linear regression model based on the partition of the cognitively normal control subjects in order to calibrate the brain-age values, and then calculated the ‘corrected’ brain-age for all study participants.

To perform complementary *post hoc* analyses, we estimated the volume of the following regions of interest: putamen and caudate volume, which were found to be associated with PD,^44^ hippocampus and total grey matter volume, which were found to be associated with AD,^45,46^ and basal forebrain volume, which was found to be reduced in both AD and PD.^44,47,48^ T_1_-weighted images were processed using the Computational Anatomy Toolbox (CAT12 v12.8, Structural Brain Mapping Group, Jena University Hospital, Germany) for SPM12, including (i) segmentation into grey matter, white matter and CSF; and (ii) high-dimensional non-linear DARTEL normalization to the MNI brain template provided by CAT12. Putamen, caudate and hippocampus volume were estimated based on the Hammers atlas.^49^ Basal forebrain volume was estimated based on the mask provided by.^50^ For statistical analysis, all volumes were normalized by total intracranial volume using proportional scaling.

### Statistical analysis

We compared demographic characteristics between diagnostic groups using Bayesian ANOVA and contingency tables as required. For these calculations, we used ‘Jeffreys’ Amazing Statistics Program’ (JASP Version 0.18.1.0), available at jasp-stats.org. We report the Bayes Factor (BF_10_) quantifying evidence against the null hypotheses.

We determined associations of the brain-age gap with chronological age, diagnosis, AD pathology markers, dopamine transporter levels, as well as motor and cognitive scores using Bayesian ANCOVA, controlling for age (except the chronological age model), sex, education and field strength. We determined the posterior estimates of the associations of interest with brain-age gap and its 90% and 95% credible intervals as primary outcomes using the library ‘brms’ in R version 4.2.1 (2022-06-23). Incidentally, many statistical programs use 89%, 90% or 95% credible interval as default. One argument against using the 95% threshold is the intention to avoid misperception of the credible interval as a binary decision tool, similar to using the *P*-value for significance testing. As stated by Mc Elreath in 2020 ‘But I don't recommend 95% intervals, because readers will have a hard time not viewing them as significance tests’, page 86 f.^51^ Here, we used both the 90% and 95% credible intervals (the default settings of brms) to underscore that the credible interval updates our knowledge on the parameter distribution after we have seen the data.

To compare fit of models with different predictors, we used Bayes factors in JASP Version 0.18.1.0. A BF_10_ of 3–10 indicates a moderate, between 10 and 30 indicates a strong, between 30 and 100 a very strong and >100 an extreme level of evidence in favour of the alternative model. In order to avoid confusion with the *P*-value, it was suggested that the Bayes factor should not be used as a binary decision threshold, but should be specified numerically and the level of evidence it conveys discussed. A shortcoming of the Bayes factor is its sensitivity to the selection of the priors^52^ (chapter 10.6, pages 292–295). Therefore, here we used the parameter estimates and their credible intervals as primary outcomes as they are relatively stable for a broad range of prior specifications and used BF_10_ only for targeted model comparisons after the parameters had been estimated. We also used sensitivity analysis for different choices of the priors to assess the stability of BF_10_ estimates.

For longitudinal data analysis, resembling a cohort study design with brain age as a continuously scaled exposure, we estimated generalized mixed effects models in a Bayesian framework with time nested within individuals with random intercept and slope terms, and longitudinal cognitive scores as outcomes. Mixed effect models assume that data were missing at random. The models contained the main effect of brain-age gap and its interaction with time, controlling for diagnosis by time, age, sex, education and field strength. We compared fit of non-Gaussian versus Gaussian models for the dependent variables using posterior predictive checks and histograms of the residuals. We determined the posterior estimates of the brain-age gap by time interaction and its 90% and 95% credible intervals as primary outcomes of this analysis. The analyses were conducted using library ‘brms’ in R version 4.2.1 (2022-06-23), accessed through R Studio.

Adherence to Bayesian Analysis Reporting Guidelines^53^ is illustrated in Supplementary Table 2.

## Results

### Demographics

As shown in Table 2, participants in the PPMI cohort differed in sex distribution and years of education, but not in age, between diagnostic groups. As expected, the groups differed in the severity of motor symptoms. In the ADNI cohort, AD patients and controls did not differ in sex distribution, age and education, but did differ in MMSE scores, as expected (Table 2).

### Cross-sectional analyses

Calendar age was associated with estimated brain age with an overall effect of Pearson's *r* = 0.823 [95% credible interval 0.803–0.840] (Supplementary Fig. 1) in the PPMI cohort. We found extreme evidence against a different age effect between the diagnostic groups (B_10_ = 1.86 ∗ 10^−7^) (Supplementary Table 3). In the ADNI cohort, calendar age was correlated with brain age as well (Pearson's *r* in AD = 0.50 [0.38–0.60], in controls = 0.71 [0.64–0.76], respectively) (Supplementary Fig. 2), and there was extreme evidence for an interaction effect of diagnosis by age (BF_10_ = 1.72 ∗ 10^5^) (Supplementary Table 4).

**Figure 1:**
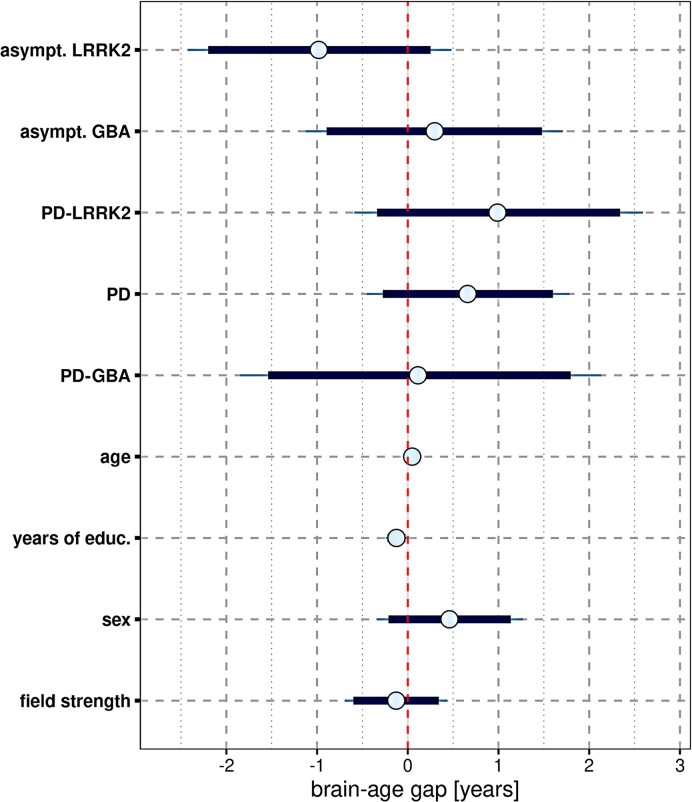
**Brain-age gap by diagnosis:** mean (circle) and 90% (thick blue line) and 95% (thin blue line) credible intervals for the effect of predictors on the brain-age gap across 1200 cases. Results of a multivariable ANCOVA model controlling for age, sex, years of education and scanner field strength. The vertical red dashed line indicates zero. PD, Parkinson's disease; LRRK2, leucine-rich repeat kinase 2 gene; GBA, glucocerebrosidase gene; asympt., asymptomatic; educ., education.

The brain-age gap for each diagnostic group in the PPMI cohort compared to controls is shown in Supplementary Table 5 and in Fig. 1, where the brain-age gap of controls equals zero. The brain-age gap was +0.7 years larger in PD cases than in controls, and −1.1 years smaller in asymptomatic LRRK2 cases than in controls. As shown in Fig. 1, however, for all group effects, the 90% credible intervals included zero. Sex had no effect on the brain-age gap, whereas longer years of education were associated with a smaller brain-age gap, indicating a younger brain age (Fig. 1). Supplementary Section 1 (page 20 ff.), including Supplementary Figs 3 and 4, reports a more in depth account of the effect of sex on brain aging in the idiopathic PD cases, including a Bayesian reanalysis of previous results.^54^ In comparison, brain-age gap in AD patients from ADNI was 5.9 [95% credible interval 4.6–7.3] years larger than in controls after controlling for calendar age, sex and field strength (Supplementary Table 6).

**Figure 2:**
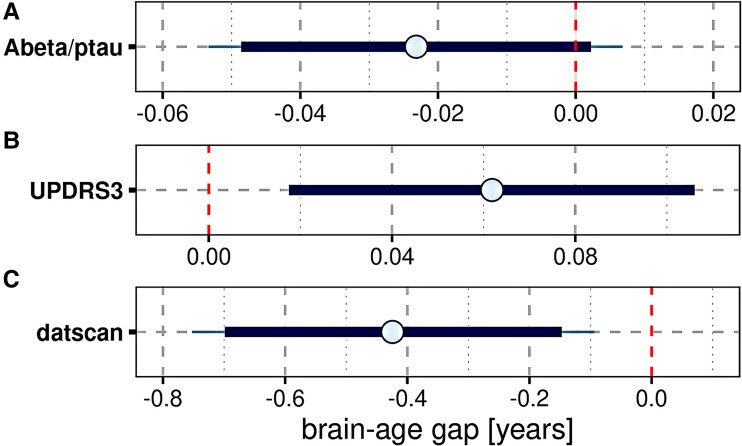
**Brain-age gap by PD and AD markers:** mean (circle) and 90% (thick blue line) and 95% (thin blue line) credible intervals for the effect of the predictors Aβ/phosphotau (**A**) (including 592 cases), UPDRS3 (**B**) (including 1191 cases) and DaTSCAN signal (**C**) (including 850 cases) on the brain-age gap. Results of a multiple regression model controlling for diagnosis, age, sex, years of education and scanner field strength. The vertical red dashed lines indicate zero. UPDRS3, Unified Parkinson's Disease Rating Scale.

When clinical and molecular markers of PD and AD were examined in the PPMI data, the following results emerged:

- A lower Aβ42/phosphotau ratio was associated with a larger brain-age gap, however, the 90% credible interval included zero (Fig. 2A). Higher motor impairment as measured by the UPDRS3 score (Fig. 2B) and lower dopamine transporter activity (Fig. 2C) were associated with a larger brain-age gap, with the 95% credible intervals excluding zero.
- For the cognitive measure, lower performance in HVLT total recall, letter–number sequencing and the Symbol-Digit Modalities Test were associated with a larger brain-age gap, with the 95% credible intervals excluding zero (Supplementary Fig. 5). MoCA score was not associated with brain-age gap (estimated effect = −0.07 [95% credible interval −0.24–0.11]).

When looking within diagnostic groups, in idiopathic PD patients, there was moderate evidence for larger brain-age gap with lower Aβ42, but not for DaTSCAN activity and phosphotau (Supplementary Table 7a). In LRRK2-PD, we found evidence for an association of larger brain-age gap with reduced DaTSCAN activity and higher phosphotau, but inconclusive evidence for Aβ42 (Supplementary Table 7b). In controls and GBA-PD patients, evidence of effects was inconclusive for all markers.

**Figure 3:**
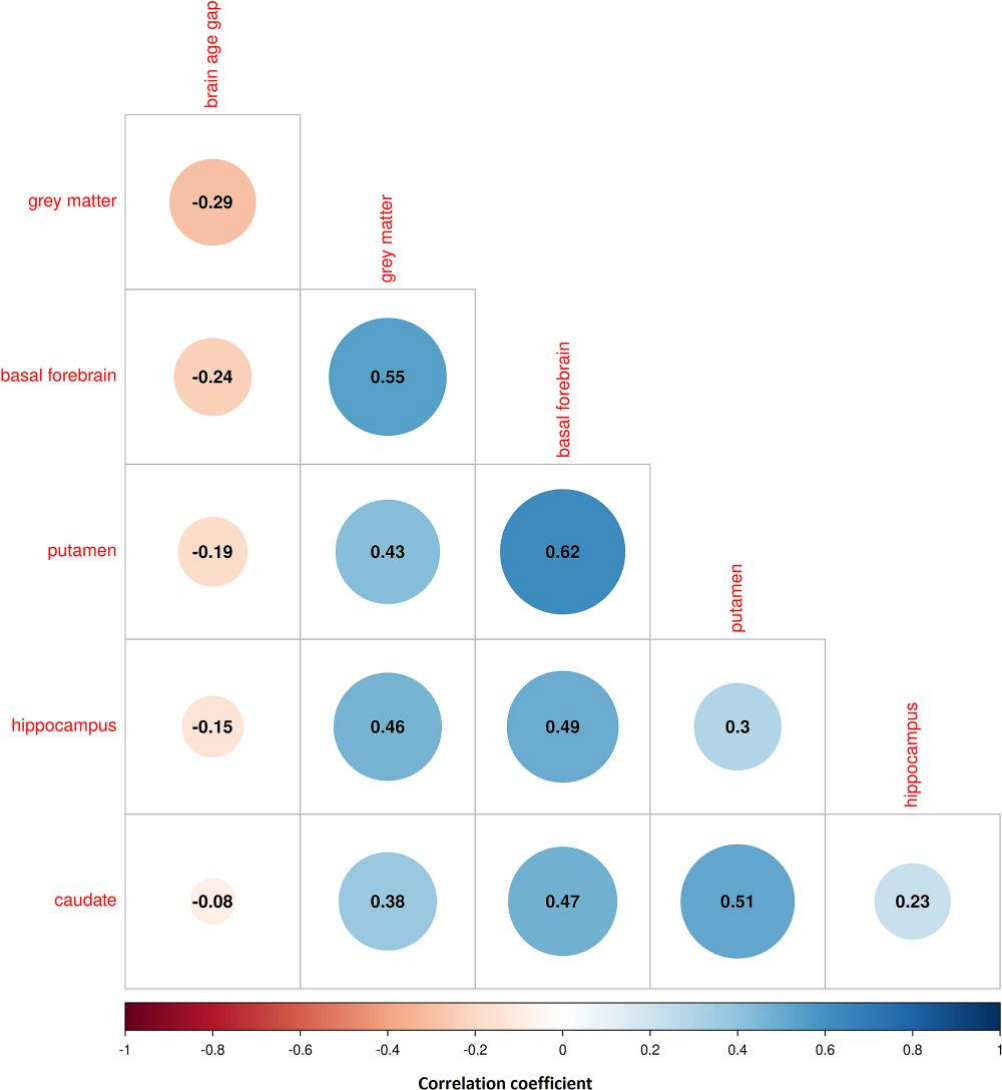
**Cross-correlations of brain-age gap and brain volumes:** cross-correlation plot of brain-age gap with brain volumes across 1200 cases. The colour scale and size of the circles indicate the size of Pearson's correlation coefficient, ranging between −1 and 1, with warm colours indicating negative and blue colours indicating positive correlations. Numbers inside the circle indicate the posterior estimate of the mean of Pearson's correlation coefficient, after controlling for diagnosis, age, sex, education, total intracranial volume and field strength, in a Bayesian correlation analysis. The 95% credible intervals of the correlation coefficients excluded zero for all correlations.

A larger brain-age gap was associated with smaller volumes in all regions considered, i.e. total grey matter, basal forebrain, putamen, hippocampus and caudate, with 95% credible intervals of the correlation coefficients excluding zero (Fig. 3, Supplementary Table 8a). When we determined model fit using leave one out cross-validation, the model fit was best for total grey matter followed by basal forebrain, putamen, hippocampus and caudate (Supplementary Table 8b). Regional volumes across diagnoses are shown in Supplementary Fig. 6A, and evidence for group differences in volumes are shown Supplementary Fig. 6B and in a searchable graph in Supplementary Materials Group Comparison of Brain Volumes file.

### Brain-age gap and longitudinal cognitive and motor scores

**Figure 4:**
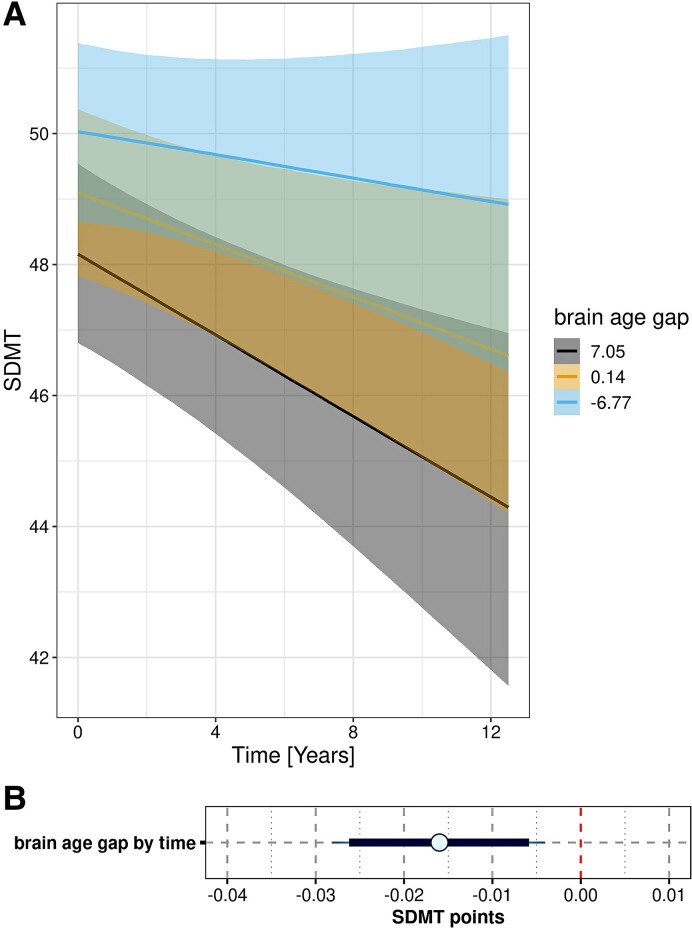
**Rate of Symbol Digits Modalities Test decline by brain-age gap:** (**A**) trajectories of change. Marginal interaction effects of time with brain-age gap on Symbol Digits Modalities Test (SDMT) decline in generalized mixed effect models predicting SDMT by brain-age gap and diagnosis and their interaction with time, controlled for age, sex, education and field strength with random slope and intercept terms for time, nested within individuals. There were 1200 cases with 6612 observations. Trajectories with 95% credible intervals are plotted for mean levels of the continuous variable brain-age gap and mean + 1 SD and mean − 1 SD (**B**) Posterior distributions of predictors. Mean (circle) and 90% (thick blue line) and 95% (thin blue line) credible intervals for the effect of predictors on the SDMT as estimated from the mixed effect regression model. The vertical red dashed line indicates zero. The lower panel zooms in on the effect of brain-age gap by time.

Mean follow-up time was 5.52 years (SD 3.68). Numbers of cases per time point are reported in Supplementary Table 9. As shown in Fig. 4 and Supplementary Fig. 7, higher brain-age gap was associated with faster decline in the Symbol-Digit Modalities Test and HVLT total recall over time, with the 95% credible intervals of the posterior parameter estimates excluding zero, and the MoCA score (Supplementary Fig. 8), with the 90% credible intervals of the posterior parameter estimates excluding zero. For motor impairment (UPDRS3) (Supplementary Fig. 9), GDS depression score, Benton Judgment of Line Orientation, semantic verbal fluency and letter–number sequencing, the 90% credible intervals for the effect of brain-age gap by time included zero. For all outcomes, except for MoCA and the Benton Judgment of Line Orientation, posterior predictive checks suggested a good fit of the data by a Gaussian model (see details in Supplementary Figs 10 and 11).

**Figure 5:**
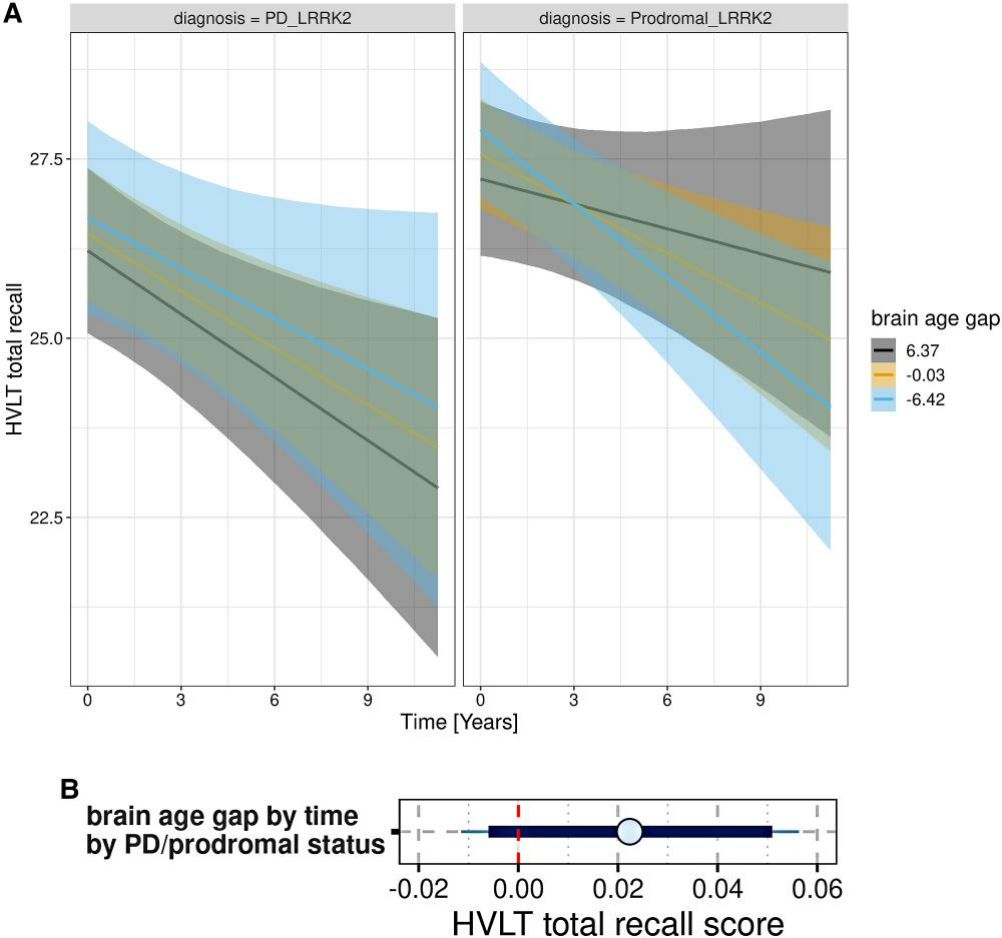
**Rate of Hopkins Verbal Learning Test total recall decline by brain-age gap by PD versus asymptomatic status in LRRK2 cases.** (**A**) Trajectories of change: marginal interaction effects of the three-way interaction effect of time by brain-age gap by prodromal versus PD LRRK2 diagnosis in generalized mixed effect models, controlled for age, sex, education and field strength with random slope and intercept terms for time, nested within individuals. There were 261 cases with 1337 observations. Trajectories with 95% credible intervals are plotted for mean levels of the continuous variable brain-age gap and mean + 1 SD and mean − 1 SD. (**B**) Posterior distributions for Hopkins Verbal Learning Test total recall score as dependent variable: mean (circle) and 90% (thick blue line) and 95% (thin blue line) credible intervals for the three-way interaction effect of brain-age gap by time by PD versus asymptomatic LRRK2 status on the Hopkins Verbal Learning Test (HVLT) total recall score as estimated from the mixed effect regression model. The vertical red dashed line indicates zero.

As an additional analysis, we determined the association of brain age with longitudinal cognitive decline within the asymptomatic and PD genetic subgroups, controlling for age, sex, education and field strength. For LRRK2, we found that the brain-age gap was positively associated with rates of decline of HVLT total recall in asymptomatic cases, i.e. a larger brain-age gap was associated with more favourable cognitive trajectories, but negatively in LRKK2-PD cases, i.e. a larger brain-age gap was associated with faster cognitive decline (Fig. 5). In contrast, in both asymptomatic and GBA-PD cases a larger brain-age gap was associated with faster decline in HVLT total recall.

## Discussion

The brain-age gap in idiopathic PD patients was only 0.7 years greater than in normal controls. Interestingly, the asymptomatic LRRK2 individuals had a 1.1 year smaller brain-age gap, whereas the asymptomatic and GBA-PD cases had unchanged brain age compared to controls. Across all cases, the brain-age gap was associated with markers of motor impairment and reduced dopamine transporter activity, but less with CSF markers of AD pathology, including Aβ42 and phosphotau. In idiopathic PD cases alone, brain-age gap was associated with Aβ42 levels, but not with dopamine transporter activity. Longitudinally, brain-age gap was associated with rates of change in episodic memory, and executive and motor function.

A brain-age gap of only +0.7 years in idiopathic PD patients is in the lower range of previous studies (summarized in Supplementary Table 1). At the same time, our analysis showed an increase in the brain-age gap of 5.9 years in the AD cases; this is consistent with previous studies reporting a brain-age gap of 5.8–10 years in AD dementia.^6,32^ The large difference in the brain-age gap between the idiopathic PD and AD cases agrees with a previous study that showed an almost 6 years higher brain age in AD compared with PD.^15^ Ran *et al*.^55^ noted that the brain age trained on healthy control data has limited specificity for certain neurodegenerative pathologies. Our comparison between PD and AD suggests that the atrophy pattern in AD represents an exacerbation of physiological brain aging, whereas the brain changes associated with idiopathic PD without dementia are only marginally captured by the brain-age algorithm.

Despite the small difference in idiopathic PD, the brain-age gap revealed interesting associations. In idiopathic PD, the brain-age gap was mainly associated with Aβ pathology rather than the degree of motor or dopamine transporter impairment. The different contributions of pathologies at different stages of PD underscore that brain age reflects a broad range of pathologies. Supporting this notion, the brain-age gap was primarily associated with global grey matter volume, which reflects a non-specific overall effect of neurodegeneration, and with the basal forebrain, which is atrophied in both PD and AD.^47,56^ Correlations of brain-age gap with PD- or AD-specific brain regions, such as the putamen or the hippocampus, were less pronounced. With respect to demographic factors, sex had no effect on brain age, but longer education was associated with a smaller brain-age gap, supporting the protective role of education on brain structure.

It is noteworthy that LRRK2 cases at the asymptomatic stage had a considerably lower brain age than controls. Preserved or even increased brain volumes at asymptomatic stages of genetic cases of neurodegenerative diseases have been reported before. Asymptomatic LRRK2 cases had increased basal forebrain, cuneus and striatum volumes, and unchanged hippocampus and thalamus volumes compared to healthy controls.^57-60^ Similarly, grey matter volume increases relative to controls have been described in the striatum of asymptomatic Parkin and PINK1 mutation carriers.^61^ These apparent volume increases in asymptomatic genetically at risk cases of PD have often been interpreted as compensatory mechanisms in the presence of preclinical dopaminergic degeneration. However, this interpretation would predict that larger brain volumes, and thus a smaller brain-age gap, would be associated with lower rates of cognitive decline. In contrast, however, in our longitudinal analysis of the asymptomatic LRRK2 cases, a smaller brain-age gap was associated with faster cognitive decline. This suggests that the relatively smaller brain-age gap in the asymptomatic LRRK2 cases represents a reactive rather than a compensatory mechanism, as the presence of this effect predicted a faster cognitive decline. The underlying nature of such reactive changes is currently unclear. In asymptomatic autosomal dominant AD cases, regional brain volume increases were found associated with neuroinflammation with glial activation and neuronal hypertrophy, which were followed by neurodegeneration and related brain atrophy only in clinical stages of autosomal dominant AD.^62^ Whether such mechanisms also play a role in asymptomatic LRRK2 cases remains to be investigated in future autopsy studies. Of note, if confirmed, our findings in asymptomatic LRRK2 cases suggest that the interpretation of the term ‘brain age’ is highly context dependent. The term brain age may be misleading when applied to disease stages where reactive brain changes with apparent volume increases rather than atrophy drive the calculation of the brain age, leading to the seemingly counterintuitive result that a younger brain age is associated with worse cognitive decline. The solution to this apparent paradox is that the algorithm does not estimate brain age in these cases, but something else.

In the idiopathic and genetic PD cases, higher brain-age gap was associated with higher rates of cognitive decline in both episodic memory and executive function measures. Association with rates of motor function decline and global cognition were less pronounced when assessing the credible intervals. Overall, the effect sizes were moderate suggesting no clear separation between different cognitive trajectories by the brain-age gap. We also found no major differences between domains more related to AD pathology, such as episodic memory, and more related to PD pathology, such as executive or motor function. This homogeneous distribution of the effect across different cognitive domains suggests that in the PD cases, the brain-age gap is a pathologically unspecific marker of overall neurodegeneration.

There are several limitations of our study. First, the data came from one single cohort, the PPMI. Our findings in idiopathic PD are consistent with findings from previous studies, which partly have been conducted in cohorts other than PPMI.^14,16,18^ The results in the genetic cases need replication in independent cohorts. Second, brain age is not a clearly operationalized concept; estimates of brain age depend on the data used to train an algorithm and on the algorithm itself. The differences between the different algorithms are substantial, and our results are in the lower range of previously reported brain-age differences in PD. To take this into account, we chose an algorithm that was already widely used^32^ and used AD data to scale our effects. Third, the PPMI cohort provides a wide range of outcomes that capture functional markers of PD, such as executive function, motor score and dopamine transporter activity, and functional and pathological markers of AD, such as episodic memory and CSF Aβ42 levels. However, the distinction between PD and AD-related outcomes is somewhat artificial, as both diseases may contribute to changes in both domains without a clear separation. At the same time, our approach acknowledges the co-pathology of AD in PD that is a relevant cofactor for the risk of cognitive decline.^63^ The findings suggest that in the clinical cases, the brain-age gap is not an appropriate measure to disentangle the contribution of AD and PD, but to capture the net effect across different pathologies. Finally, conclusions about the underlying mechanisms for previously unexpected effects, such as the inverse association of brain-age gap with cognitive decline in asymptomatic versus LRRK2-PD cases, were drawn *post hoc* and could not be tested confirmatively.

In summary, consistent with our expectation, we found higher brain-age gap in idiopathic PD, but effects were small. Brain age was most closely correlated with global grey matter volume, but less with basal ganglia and hippocampus volume. This indicates that cortical atrophy was a main driver of brain age and it explains why brain age changes were less pronounced in PD than in AD cases. This limits the utility of the brain-age algorithm to serve as a biomarker in PD. We confirmed an association of brain-age gap with cognitive, motor and functional markers of PD, but also with functional and pathological markers of AD, particularly when focusing on cases with idiopathic PD, where the brain-age gap was better explained by markers of AD pathology than the degree of PD-related changes in motor and dopaminergic function. The variation of brain age within the idiopathic PD cases had some predictive value for rates of cognitive decline, however, brain age may not be the best single marker of brain pathology in PD. Unexpectedly, we found lower brain-age gap in asymptomatic LRRK2 cases. This indicates that the interpretation of the brain age depends on the actual context of use. What the brain-age algorithm measures in asymptomatic LRRK2 may not really be related to something like age of the brain, since brain age in these individuals was seemingly younger than in age-matched controls. The results in the asymptomatic LRRK2 cases with poorer cognitive outcome in people with a younger brain age give rise to the hypothesis that the brain-age algorithm here does not detect an effect of atrophy but possibly reactive effects to preclinical accumulation of pathology. It is important to keep in mind that the results of brain age analyses vary with the different algorithms used. Other groups using different algorithms yielded larger brain-age gaps, but still far below those seen in AD.

## Supplementary Material

fcae382_Supplementary_Data

## Data Availability

This analysis used data openly available from PPMI and ADNI. Data were obtained on 6 March 2023 from the Parkinson's Progression Markers Initiative (PPMI) database (www.ppmi-info.org/access-dataspecimens/download-data), RRID:SCR_006431, and the ADNI database (https://adni.loni.usc.edu). For up-to-date information on the PPMI study, visit www.ppmi-info.org. Sample code for the longitudinal analysis in R is provided in Supplementary Materials Example Codes. The brain age estimates will be made available by request from any qualified investigator through the corresponding author.
