# Supplementary material for: Brain age in genetic and idiopathic Parkinson's disease
Source: Brain Commun. 2024 Dec 20;6(6):fcae382. doi: 10.1093/braincomms/fcae382 (PMC11660940; doi:10.1093/braincomms/fcae382)
Supplement: fcae382_Supplementary_Data [file fcae382_supplementary_data.zip › Supplementary Materials.pdf]

Supplementary Material

Supplementary Table 1: Overview of Previous Studies on Brain Age in Parkinson's disease

Authors	year	diagnostic groups	brain age algorithm	research questions	key findings	further comments
**Eickhoff et al.** [2]	2021	**372** idiopathic PD (304 from PPMI, 68 local sample) **172** HC (101 from PPMI, 71 local sample)	multivariate machine learning model trained on an independent, multi-site reference sample (training set n = 3960 subjects). Ensemble of linear support vector regression (SVR) with stratified subsampling. SVR models was performed using LibSVM toolbox	Assessment of (univariate) GMV and brain age differences between PD and controls; relationship between brain age and clinical impairment (UPDRS-III, MoCA), disease duration and severity in Parkinson's disease	BAG: + 2,9 years (PD local cohort: +3,3 years, PD PPMI-cohort: +2,8) years; evident correlation between MoCA scores and BAG (r = 0.19, p<0.001), between motor symptoms (UPDRS-III score) and BAG (r = 0.14, p<0.006) and between disease duration and BAG (r = 0.14, p<0.008); PD patients showed widespread cortical atrophy but only a moderate (r = 0,22) correlation between parcel-wise atrophy estimates of	Compares newly diagnosed (PPMI cohort) and more chronic PD patients (local sample) with a healthy control group

Authors	year	diagnostic groups	brain age algorithm	research questions	key findings	further comments
					the (early stage) PPMI cohort and the (more advanced) local cohort	
**Beheshti et al.** [3]	2020	**160** PD (PPMI)<br>**129** AD (ADNI)	Support vector regression (SVR) algorithm implemented in LIBSVM library with a linear kernel and default settings (training set n = 839 healthy individuals) using GM and WM model	differences in Brain-EAD between AD and PD; following hypotheses were assessed:<br>a) PD patients have a higher WM Brain-EAD than GM Brain-EAD.<br>b) There are significant Brain-EAD differences between PD and AD patients.<br>c) AD patients have a significantly "older-appearing"	Brain-EAD:<br>**PD**: GM +1,5 ± 6,03 years<br>WM +2,47 ± 5,85 years;<br>**AD**: GM +9,29 ± 6,43 years,<br>WM +8,85 ± 6,62 years<br><br>**PD**: Brain-EAD shows significant relationship with MoCA, MDS-UPDRS Part I, and UPSIT - Total Score (r = –0.15, r = 0.21, and r = –0.14, respectively; $p < 0.05$)<br>no significant relationship between Brain-EAD and disease	No significant correlation was found between Brain-EAD and GDS, MDS UPDRS Total, SBRs of the left/right putamen and caudate

Authors	year	diagnostic groups	brain age algorithm	research questions	key findings	further comments
				brain compared with PD patients. (Hypotheses are original from paper)	duration and MDS-UPDRS Part III.	
**Charissé et al.** [4]	2022	LANDSCAPE cohort<br>Baseline:<br>**178** PD (83 PD-NC, 78 PD-MCI, 17 PD-D),<br>**84** HC<br><br>Follow up:<br>**137** PD (61 PD-NC, 62 PD MCI, 14 PD-D),<br>**67** HC	estimate of brain age based on brain atrophy termed “Spatial Pattern of Atrophy for Recognition of Brain Age” (SPARE-BA) -> support vector re-gression function from the Scikit-Learn library with a radial basis function kernel, study collective: 8287 healthy individuals	Hypotheses :<br>a) Association of SPARE-AD score and increased RBA with dysfunction in different cognitive domains in PD (original from paper: “Are SPARE-AD scores and increased RBA in PD associated with dysfunction in different cognitive domains?”)	(a) PD-MCI and PD-D had increased SPARE-AD scores compared to PD-NC and HC<br>(b) RBA was increased in all PD groups: +2.2 years in non-demented PD, +3.5 years in patients with PD-D<br>(c) Elevated SPARE-AD scores were associated with impairment in executive, memory, and language functions, whereas increased RBA correlated with	Uses “relative brain age” (RBA) as brain age gap

Authors	year	diagnostic groups	brain age algorithm	research questions	key findings	further comments
				b) Association of increased brain age gap and patients´ current cognitive status or future status conversion in PD (original from paper: “Do increased brain age gaps in PD relate to the patients’ current cognitive status or future status conversion?”)	dysfunction in the attention and working memory domain, and with motor disease severity.<br>(d) PD-NC to PD-MCI converters had increased baseline RBA and SPARE-AD scores as compared to patients with stable PD-NC<br>(key findings already summarized in paper, slightly modified)	
**Chen et al.** [5]	2022	Local cohort<br>**23** MSA<br>**33** PD<br>**34** HC	GM-based and WM-based age prediction model; employed a 12-layer feed-forward cascade neural network architecture to predict brain age	Difference in brain age between MSA and PD<br>Identification of image features that underlie the	GM-PAD:<br>MSA +9,33 years,<br>PD +0,75 years,<br>HC -1,47 years<br>WM-PAD:<br>MSA +9,27 years,<br>PD +1,9 years,	Explains brain age algorithm in another paper in more detail [6]

Authors	year	diagnostic groups	brain age algorithm	research questions	key findings	further comments
			(training set: 482 cognitively normal participants)	aging variation between MSA and PD	HC -0,79 years<br>markedly different neuroanatomical contributions to brain aging between MSA and PD patients	

AD = Alzheimer's disease, BAG= brain age gap, Brain-EAD = brain-estimated age difference, GDS = Geriatric Depression Scale, GM(V) = grey matter (volume) HC = Healthy control, MCI = mild cognitive impairment, MDS-UPDRS = Movement Disorder Society-Sponsored Revision of the Unified Parkinson's Disease Rating Scale, MSA = multiple system atrophy, NC = normal cognition, PAD = predicted age difference, PD-(D) = Parkinson's disease (with dementia), RBA = relative brain age, SPARE-BA = spatial pattern of atrophy for recognition of brain age, WM = white matter

Supplementary Table 2: Checklist of Bayesian Analysis Reporting Guidelines[7]

Preamble

A. *Why Bayesian. If the audience requires it, explain what benefits will be gleaned by a Bayesian analysis (as opposed to a frequentist analysis).*

   The rationale for using a Bayesian approach is outlined in the last paragraph of the introduction.

B. *Goals of analysis. Explain the goals of the analysis. This prepares the audience for the type of models to expect and how the results will be described.*

   The analysis goals are reported in the last paragraph of the introduction.

Step 1. Explain the model

A. *Data variables. Explain the dependent (predicted) variables and independent (predictor) variables.*

   Dependent and independent variables are explained in the methods section

B. *Likelihood function and parameters. For every model, explain the likelihood function and all the parameters, distinguishing clearly between parameters of primary theoretical interest and ancillary parameters. If the model is multilevel, be sure that the hierarchical structure is clearly explained, along with any covariance structure if multivariate parameter distributions are used.*

   In the statistics section, we describe the model construction. For the cross-sectional analysis, the parameters of primary interest were chronological age, diagnosis, markers of AD pathology, cognitive function, motor function, and dopamine transporter activity. The models were controlled for effect of age (except the chronological age model), sex, education, and field strength. For the longitudinal analyses, the primary effect of interest was the interaction of brain-age gap by time, controlling for age, sex, education, and field strength. We used a multilevel structure with random intercept and slope, nested within individuals. Distribution assumptions were checked by posterior predictive checks, and in the

case of MoCA scores, the histograms of the residuals under different distribution assumptions, as shown in Supplementary Figures 8 and 9.

C. *Prior distribution. For every model, explain and justify the prior distribution of the parameters in the model.*

For the ANCOVA analyses, we used JASP's default Jeffreys-Zellner-Siow (JZS) prior with an r scale of 0.354. For the mixed effect models, we used brms default flat priors. In a sensitivity analysis, we used Student's t distribution with mean 0, standard deviation 100 and one degree of freedom. Parameter estimates were widely unaffected by this alternative prior choice.

D. *Formal specification. Include a formal specification (mathematical or computer code) of the likelihood and prior, located either in the main text or in in publicly and persistently accessible online supplementary material.*

For example, for HVLT total recall (HVLT_tr) score as an outcome the **likelihood**, i.e. the data generating process of the outcome, was defined as:

HVLT_tr ~ time:diagnosis + time:brainage_gap + time + brainage_gap + diagnosis + age + sex + education + Fieldstrength (1 + time|Subject_ID),

with a Gaussian distribution for HVLT_tr scores, and flat priors for the fixed effect parameters.

Step 2. Report details of the computation

A. *Software. Report the software used, including any specific added packages or plugins.*

The software used and specific packages are reported in the statistics section.

B. *MCMC chain convergence. Report evidence that the chains have converged, using a convergence statistic such as PSRF, for every parameter or derived value.*

MCMC chain mixing was checked using trace plots in R (brms::plot), Rhat[8] was used for all parameters and models calculated.

C. *MCMC chain resolution. Report evidence that the chains have high resolution, using the ESS, for every parameter or derived value.*

   For the longitudinal models, we used four chains with 8,000 iterations each, including 2,000 warm-up iterations. The bulk and tail effective sample size (ESS) was higher than 6,000 and 12,000, respectively, for all parameters in the model, indicating low autocorrelation of the MCMC chains.

Step 3. Describe the posterior distribution

A. *Posterior predictive check. Provide a posterior predictive check to show that the model usefully mimics the data.*

   Results of posterior predictive checks are summarized in the results section, and in Supplementary Figures 8 and 9.

B. *Summarize posterior of variables. For continuous parameters, derived variables and predicted values, report the central tendency and limits of the credible interval. Explicitly state whether you are using density-based values (mode and HDI) or quantile-based values (median and ETI), and state the mass of the credible interval (for example, 95%).*

   Posterior variables are reported as mean and 90% and 95% credible intervals, including the parameters of interest for the longitudinal models.

C. *BF and posterior model probabilities. If conducting model comparison or hypothesis testing, report the BF and posterior probabilities of models for a range of prior model probabilities.*

   For the cross-sectional analyses, we report Bayes Factors comparing the index model with the null model, including the confounder variable age, sex, education, and field strength. For the longitudinal data analysis, we abstained from reporting Bayes factors as they have been reported to be highly dependent on the choice of the prior choice for mixed effect models, whereas parameter estimates are robust for different priors. In sensitivity analyses, we checked the stability of Bayes factors and parameter estimates with different priors in mixed

effect regression models (cf. section 1C). We found large differences in Bayes factors between a flat prior and a prior distributed according to a Student's t distribution with mean 0, standard deviation 100 and one degree of freedom, whereas parameter estimates were stable.

Step 4. Report decisions (if any) and their criteria

A. *Why decisions? Explain why the decisions are theoretically meaningful and which decision procedure is being used. Regardless of which decision procedure is used, if it addresses null values, it should be able to accept the null value not only reject it.*

   Decisions were based on the Bayes factor of the index model compared with the null model for the cross-sectional data. We reported the Bayes factor value and discussed the level of evidence it conveyed to avoid a misconception of the Bayes factor as some type of significance testing. Further we considered 90% and 95 credible intervals for parameter estimates.

B. *BF, decision threshold and model probabilities. If using model comparison or hypothesis testing as the basis for a decision, state and justify the decision threshold for the posterior model probability, and the minimum prior model probability that would make the posterior model probability exceed the decision threshold.*

   See Point A.

C. *Estimated values too. If deciding about null values, always also report the estimate of the parameter value (central tendency and credible interval).*

   See point A.

Step 5. Report sensitivity analysis

A. *For default priors. If using a default prior, show the effect of varying its settings. Be sure that the range of default priors constitutes theoretically meaningful priors, and consider whether they mimic plausible empirically informed priors.*

By default, brms uses flat priors for population level (fixed) effect parameters. In a sensitivity analysis, we repeated the analyses, using very weakly informed priors, with a Student's t distribution of mean 0 and standard deviation of 100 and one degree of freedom. See section 1C.

Step 6. Make it reproducible

A. *Software and installation. Explain all the software that is necessary and where to obtain it. If possible, use non-proprietary software.*

   We used R and JASP as free to use software with specification of version and libraries used as detailed in the statistics section. We also added the supplementary file 3, providing code for an exemplary longitudinal data analysis.

B. *Software version details. The posted script should include detailed information about the software version numbers*.

   See point A.

Supplementary Table 3: Effect of age with and without diagnosis in the PPMI cohort

Models	P(M)	P(M|data)	BF_M	BF_{10}	error %
Null model (incl. diagnosis)	0.333	1.44×10^{-291}	2.89×10^{-291}	1.000	
Age	0.333	1.00	$1.08\times10^{+7}$	$6.93\times10^{+290}$	1.17
Age + Age*diagnosis	0.333	1.86×10^{-7}	3.72×10^{-7}	$1.29\times10^{+284}$	4.58

P(M) - prior model probability

P(M|data) - model probability given the data

BF_M - degree to which the data have changed the prior model odds

BF_{10} - Bayes factor in favor of the alternative model

error % - error estimate of the routine used by the R "BayesFactor" (https://cran.r-project.org/web/packages/BayesFactor/index.html) package for the computation of the Bayes factor

Bf_{10} for an additional effect of age* diagnosis was $1.29\times10^{+284}/6.93\times10^{+290} = 1.86*10^{-7}$

Supplementary Table 4: Effect of age with and without diagnosis in the ADNI cohort

Models	P(M)	P(M|data)	BF_M	BF_{10}	error %
Null model (incl. diagnosis)	0.333	1.71×10^{-48}	3.43×10^{-48}	1.000	
Age	0.333	5.81×10^{-6}	1.16×10^{-5}	$3.39\times10^{+42}$	11.59
Age + Age*diagnosis	0.333	1.00	$3.4\times10^{+5}$	$5.83\times10^{+47}$	1.03

P(M) - prior model probability

P(M|data) - model probability given the data

BF_M - degree to which the data have changed the prior model odds

BF_{10} - Bayes factor in favor of the alternative model

error % - error estimate of the routine used by the R "BayesFactor" (https://cran.r-project.org/web/packages/BayesFactor/index.html) package for the computation of the Bayes factor

Bf_{10} for an additional effect of age* diagnosis was $5.83\times10^{+47}/3.39\times10^{+42} = 1.72*10^5$

Supplementary Table 5: Brain-age gap in the PPMI cohort

	Mean [years] (SD)	min-max [years]
**Controls**	0 (6.9)	-25.1 - +15.6
**PD**	0.7 (7.9)	-21.7 - +17.7
**PD-GBA**	0.07 (6.7)	-16.3 - +15.6
**PD-LRRK2**	1.2 (5.9)	-12.5 - +15.4
**Asymptomatic GBA**	0.023	-30.2 - +15.0
**Asymptomatic LRRK2**	-1.1 (6.5)	-15.2 - + 15.5

Supplementary Table 6: Brain age gap in the ADNI cohort

	Mean [years] (SD)	min-max [years]
**Controls**	0 (6.7)	-38.3 - +17.9
**AD**	6.2 (7.9)	-28.5 - +24.7

Supplementary Table 7: Within group results for DatScan and AD pathology markers

Supplementary Table 7a: Idiopathic PD

**Effects**	**P(incl)**	**P(excl)**	**P(incl|data)**	**P(excl|data)**	**BF_{incl}**
DaTScan activity	0.500	0.500	0.296	0.704	0.421
Abeta42	0.500	0.500	0.744	0.256	2.904
pTau	0.500	0.500	0.271	0.729	0.371

Supplementary Table 7b: PD-LRRK2

**Effects**	**P(incl)**	**P(excl)**	**P(incl|data)**	**P(excl|data)**	**BF_{incl}**
DaTScan activity	0.500	0.500	0.890	0.110	8.130
Abeta42	0.500	0.500	0.439	0.561	0.784
pTau	0.500	0.500	0.880	0.120	7.305

Supplementary Table 8: Brain age gap and TIV normalized volumes brain volumes

Supplementary Table 8a: Correlations controlled for age, sex, education, field strength, and TIV

**Brain age gap with …**	**partial r**	**95% CI**	**BF_{10}**
grey matter	-0.29	-0.34 - -0.24	$2.5*10^{22}$
basal forebrain	-0.24	-0.29 - -0.18	$5.2*10^{13}$
putamen	-0.19	-0.24 - -0.13	$2.7*10^{8}$
hippocampus	-0.15	-0.21 - -0.10	$4.2*10^{4}$
caudate	-0.08	-0.14 - -0.03	3.8

Supplementary Table 8b: Model fit (leave one out cross-validation)

	Elpd(diff)	SE(diff)
Grey matter	0.0	0.0
Basal forebrain	-19.6	11.1
Putamen	-31.9	11.8
Hippocampus	-40.7	12.4
Caudate	-50.2	12.3

Supplementary Table 9: Number of cases per time point

Time points (approx. 1 year apart)	Number of cases
1 (baseline)	1200
2	1102
3	920
4	849
5	768
6	640
7	466
8	352
9	274
10	211
11	124
12	35
13	2

Supplementary Figure 1: Association of brain age with calendar age according to diagnoses in the PPMI cohort

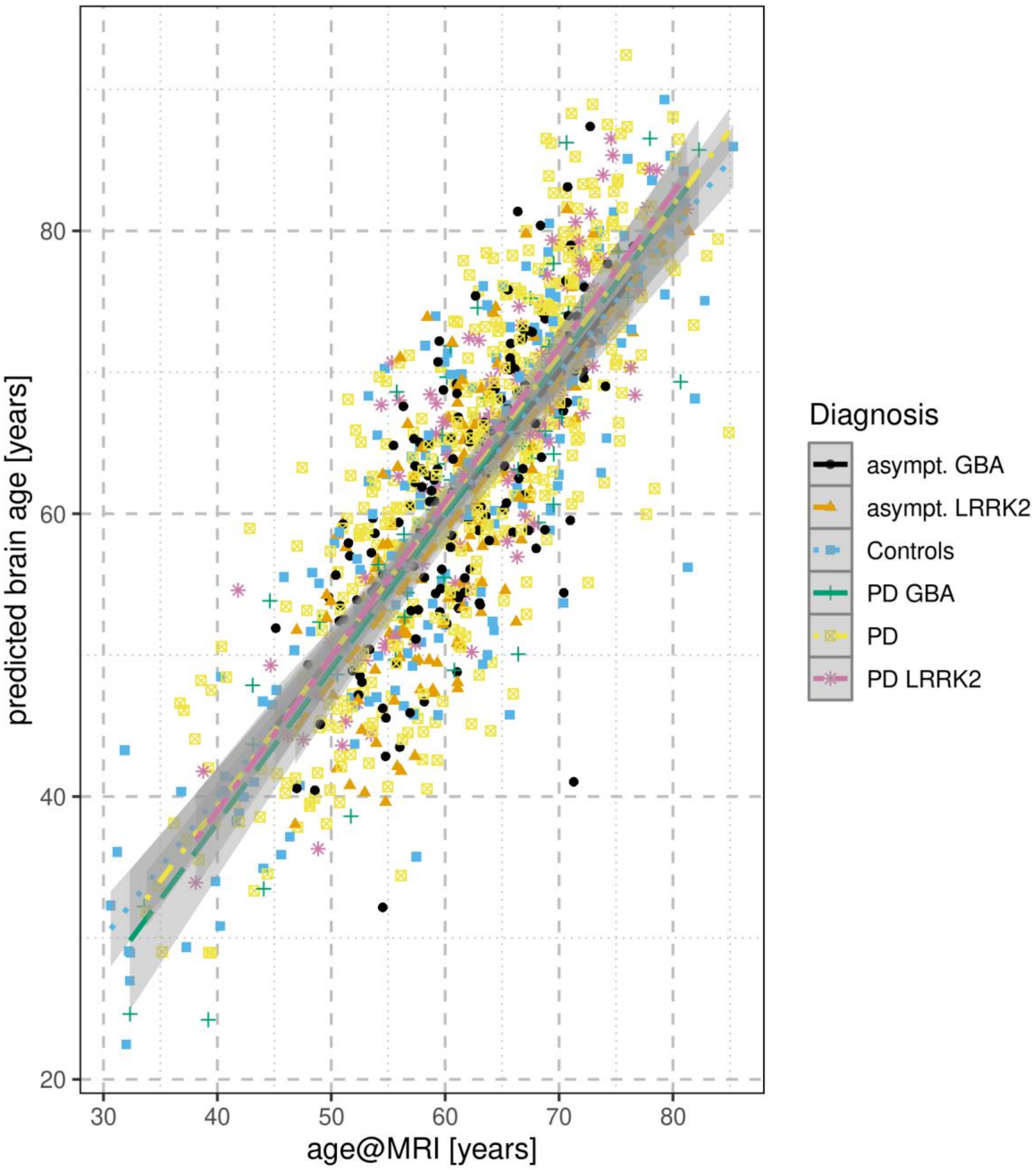

Scatter plot of predicted brain age by chronological age using linear least square regression across N= 1,200 cases, split according to diagnoses with group specific regression lines and grey shaded 95% credible intervals.

Supplementary Figure 2: Association of brain age with calendar age according to diagnoses in the ADNI cohort

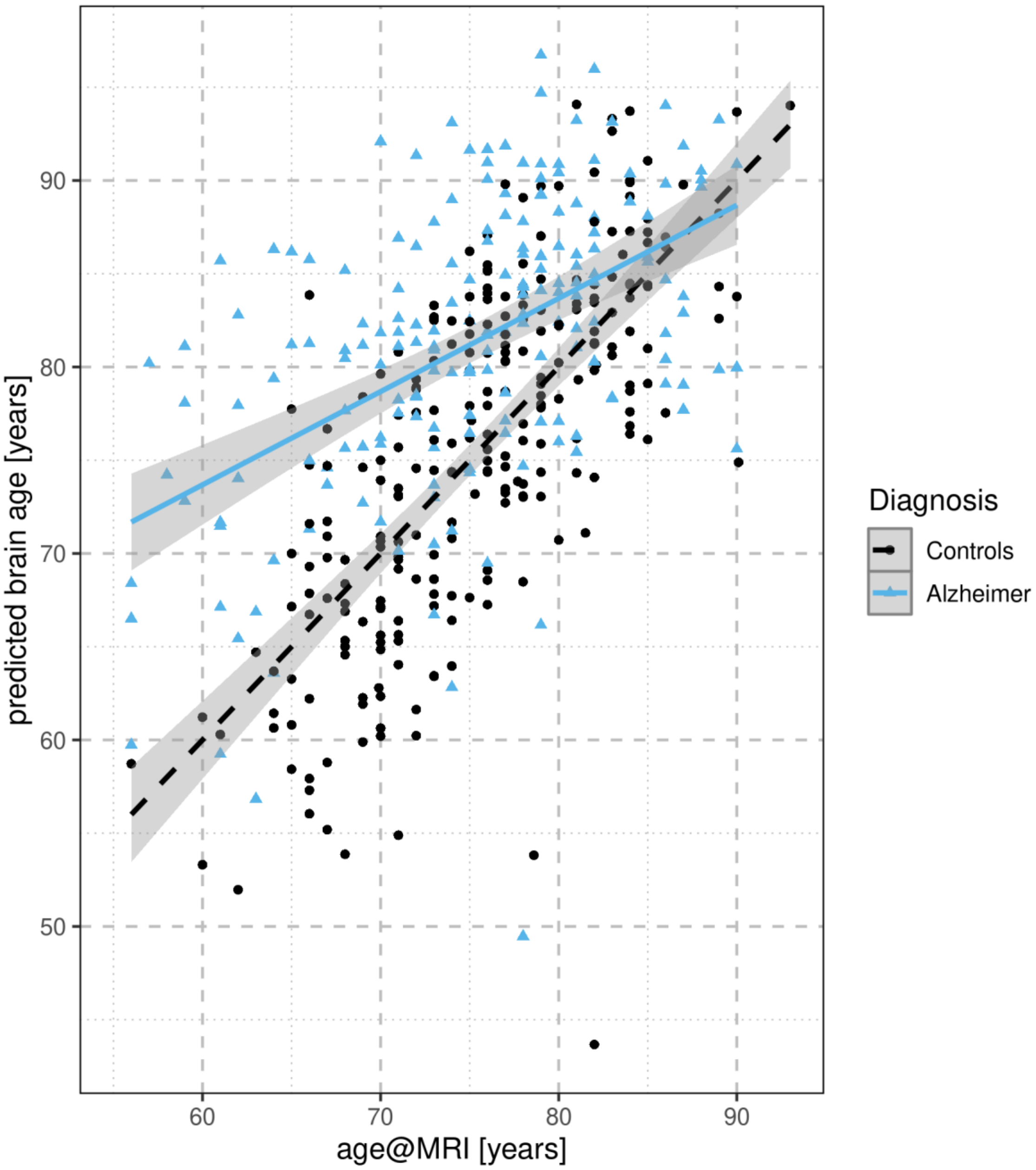

Scatter plot of predicted brain age by chronological age using linear least square regression across N= 441 cases, split according to diagnoses with group specific regression lines and grey shaded 95% credible intervals.

Supplementary Section 1 - effect of sex on brain-age gap in idiopathic PD cases

A previous study [1] in a subsample of 373 idiopathic PD cases from the PPMI cohort found a significant effect of sex on the estimated brain-age gap, with lower brain age in women than in men. Here, we replicated the previous analysis using a Bayesian approach and compared results between the previous report and our idiopathic PD sample. Of note, both samples come from the same cohort so that we assume that the previous sample [1] was more or less a subset of the current sample.

Extending this previous analysis, we conducted two additional analyses in the idiopathic PD cases:

- We reanalyzed the results of [1], using a Bayesian independent t-test in JASP.
- We determined the sex effect with and without controlling for covariates in the idiopathic PD cases from our sample. The analysis without controlling for covariates replicated the analyses of [1] in their full PD sample, the analysis with confounding covariates replicated the analysis of [1] in their propensity-matched subsample of PD cases.

Ad 1): The effect size estimate from [1] was T = 2.5, with n_{female} = 129, and n_{male} = 129. Here, the male PD sample was propensity score-matched to the female PD cases for age, education, motor impairment and cognition. The resulting Bayes factor in favor of a sex difference BF_{10} was 2.6, indicating an anecdotal level of evidence for a sex effect. When considering the non-matched PD samples from [1], the effect size T was 2.26, with n_{female} = 129, and n_{male} = 244, the resulting BF_{10} was 1.37, indicating inconclusive evidence for a sex effect.

Ad 2): When we considered the effect of sex in the idiopathic PD cases of our sample without any covariates, the BF_{10} was 1.29, very similar to the result of [1] in the not matched sample. The sequential analysis, shown in Figure A below, suggests that effects vary with increasing number of cases, but basically remain at an anecdotal level of evidence.

Supplementary Figure 3: Sequential analysis of sex effect on brain-age gap in idiopathic PD cases

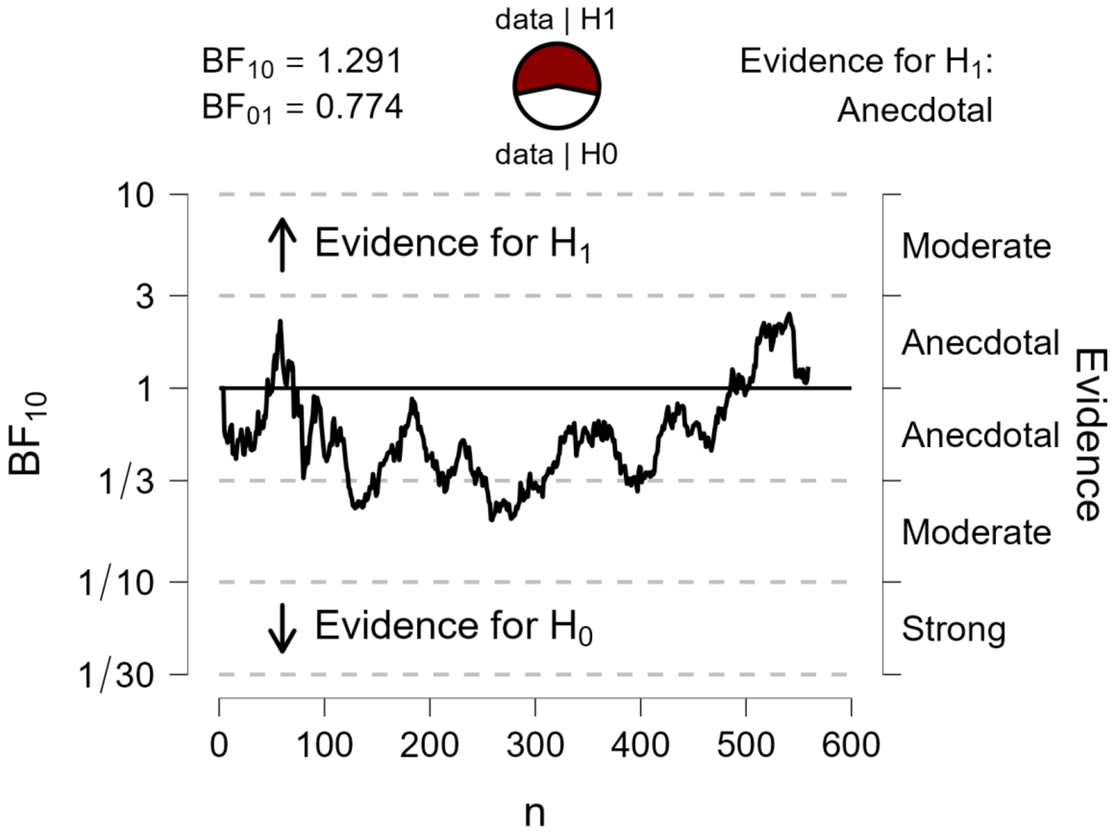

As number of cases increases, levels of evidence for an effect vary, but remain at an anecdotal level of evidence in either direction.

When we considered the effect of sex, taking age, education, and field strength into account, the BF_{10} was 1.48, consistent with inconclusive evidence for a sex effect.

Thus, the results are consistent between the previous study and our analysis (including partially overlapping samples): the level of evidence for an effect of sex was anecdotal at best. This suggests that if there is a sex effect, it is very small. This is also illustrated by the plot of the posterior distributions of the brain-age gap in female and male idiopathic PD cases (Figure B below), which largely overlap.

Supplementary Figure 4: Brain-age gap in female and male patients with idiopathic PD

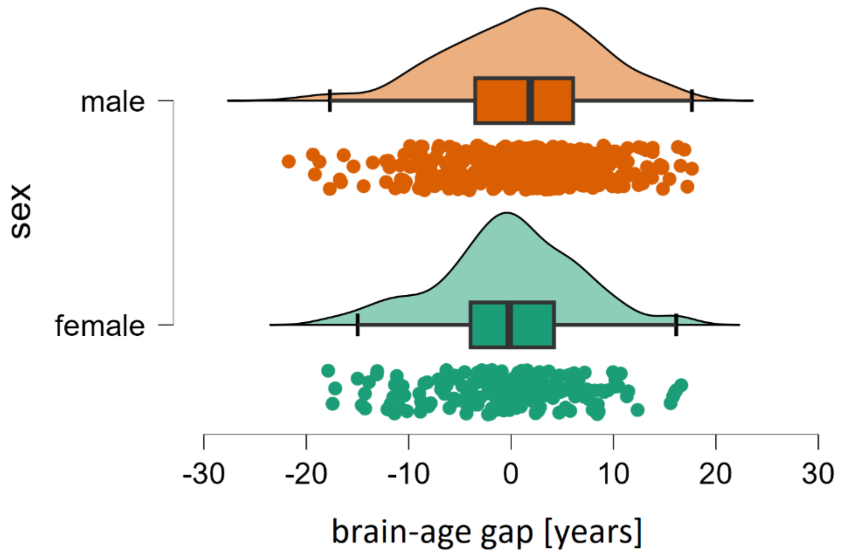

Posterior distribution of brain age gap in female and male patients with idiopathic PD

Supplementary Figure 5: Brain-age gap by cognitive score

Supplementary Figure 5a: HVLT total recall

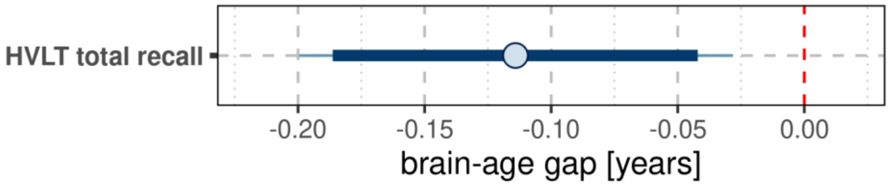

Supplementary Figure 5b: LNS

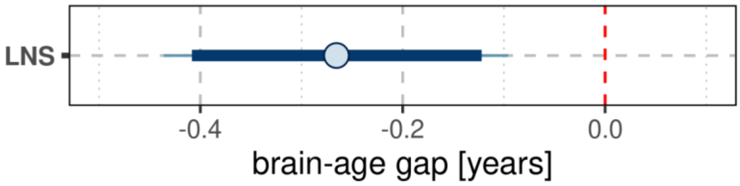

Supplementary Figure 5c: SDMT

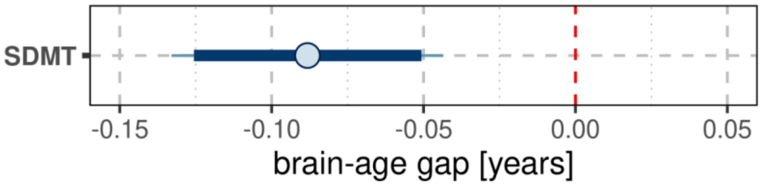

Mean (circle) and 90% (thick blue line) and 95% (thin blue line) credible intervals for the effect of the predictors Hopkins Verbal Learning Test (HVLT) total recall (Supplementary Figure 5a), Letter number Sequencing (LNS) (Supplementary Figure 5b), and Symbol-Digit Modalities Test (SDMT) (Supplementary Figure 5c) on the brain-age gap. Results of a multiple regression model controlling for diagnosis, age, sex, years of education, and scanner field strength across 1,200 cases. The vertical red dashed line indicates zero.

Supplementary Figure 6a: Brain volumes by diagnoses

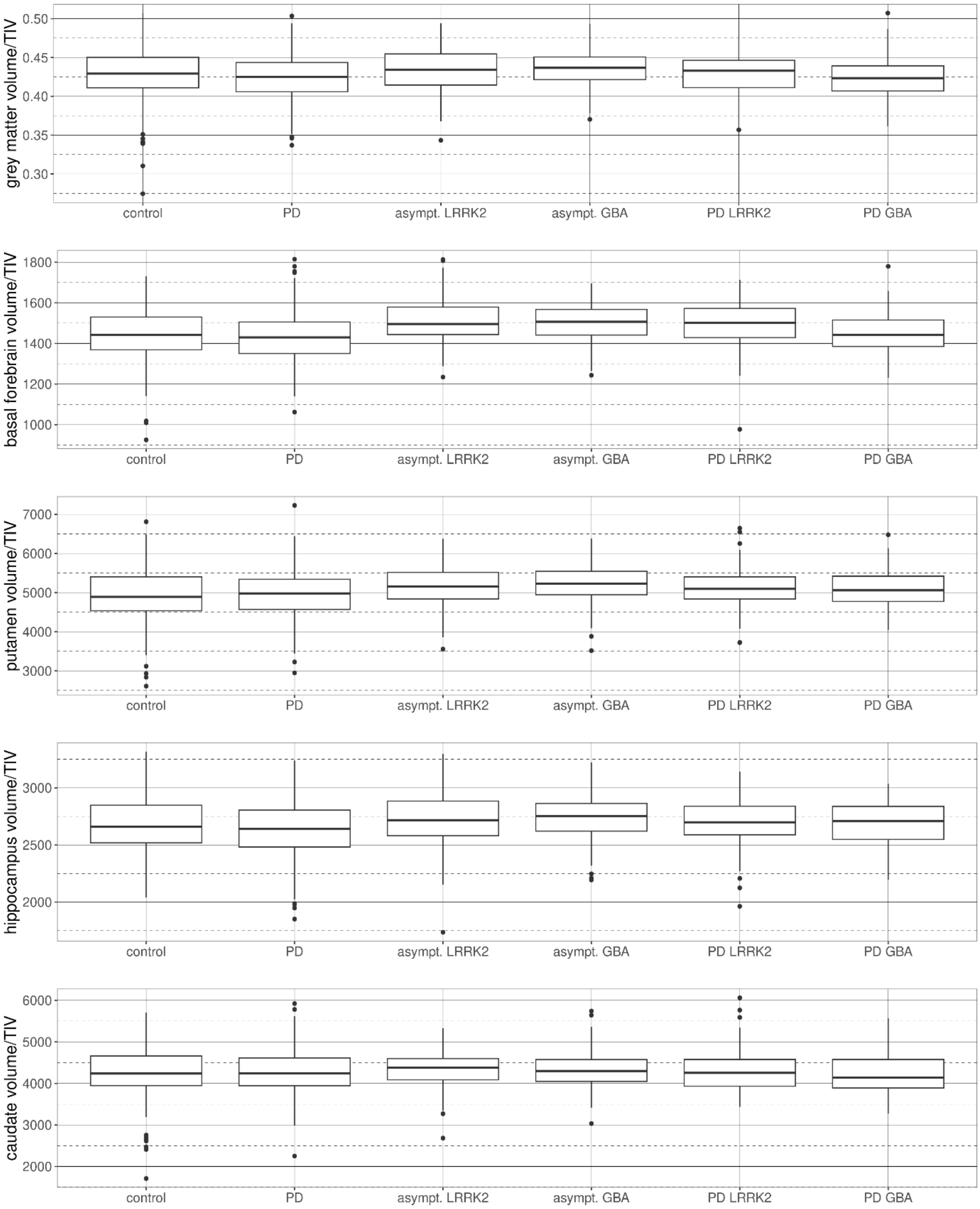

Boxplots of brain volumes by diagnoses. All volumes have been normalized to total intracranial volume.

Supplementary Figure 6b: Heatmap of post hoc Bayes factors for group differences

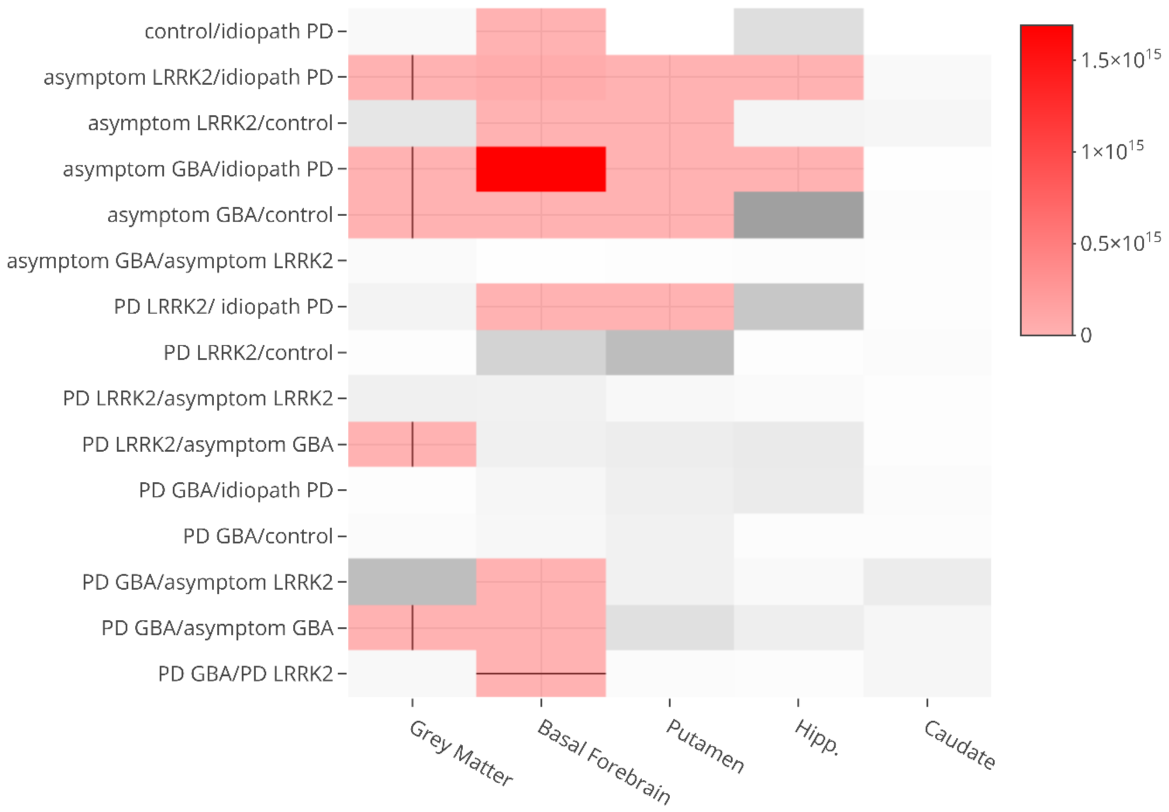

Heatmap of the Bayes factors in favor of a group difference (BF_{10}) of post hoc group comparisons following an ANCOVA analysis across all groups. Comparisons with a BF_{10} above 3, indicating at least moderate level of evidence for a group difference are in red, cells below this value in grey. An interactive version of this heatmap where cell values can be highlighted is appended as "Supplementary Materials Group Comparison of Brain Volumes" file.

Supplementary Figure 7: Rate of Hopkins Verbal Learning Test total recall decline by brain-age gap

Supplementary Figure 7a: Trajectories of change

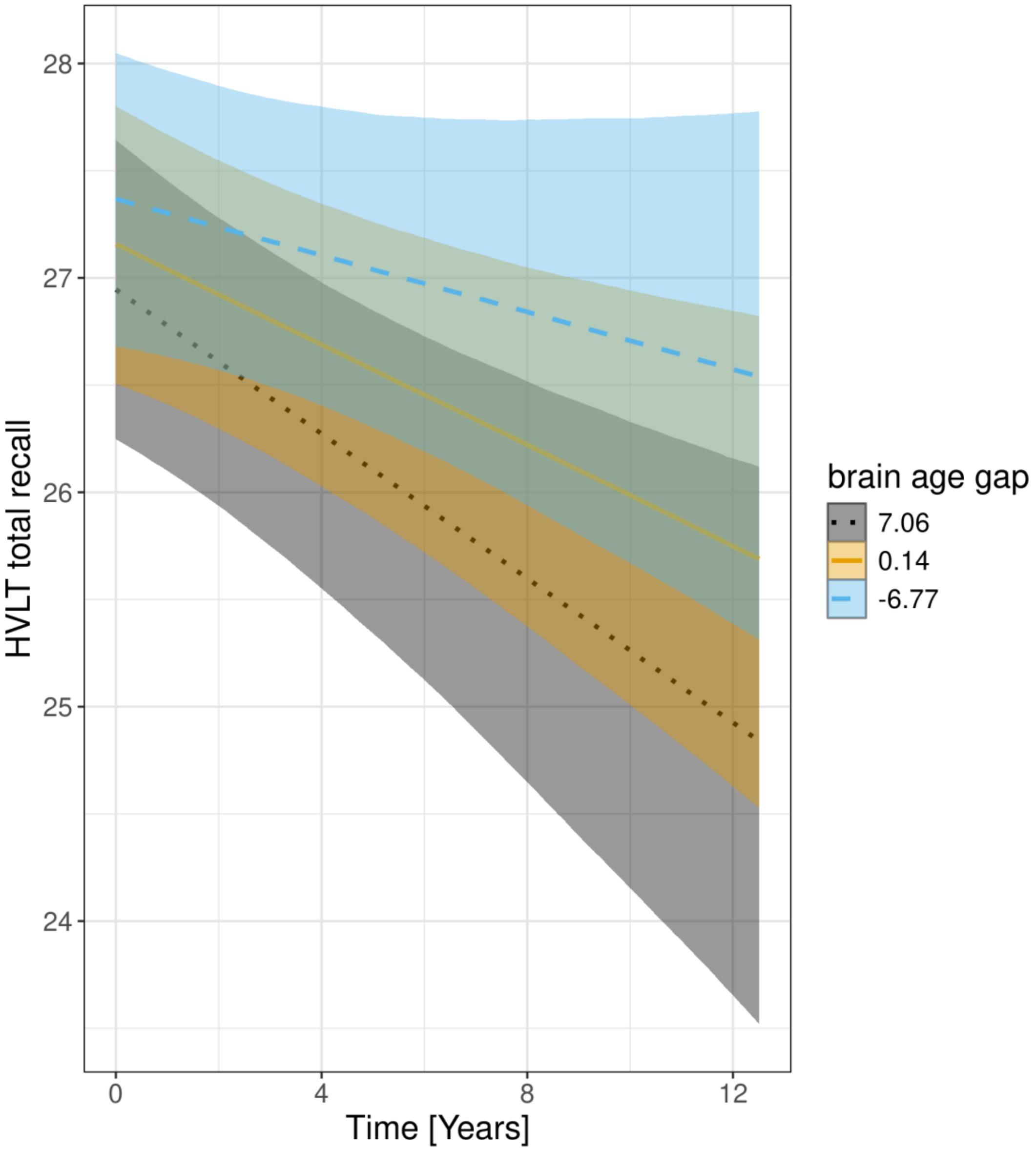

Marginal interaction effects of time with brain-age gap on Hopkins Verbal Learning Test (HVLT) total recall decline in generalized mixed effect models predicting HVLT total recall by brain-age gap and diagnosis and their interaction with time, controlled for age, sex, education, and field strength with random slope and intercept terms for time, nested within individuals. There were 1,200 cases with 6,618 observations. Trajectories with 95% credible intervals are plotted for mean levels of the continuous variable brain-age gap and mean + 1 standard deviation and mean – 1 standard deviation.

Supplementary Figure 7b: Posterior distribution

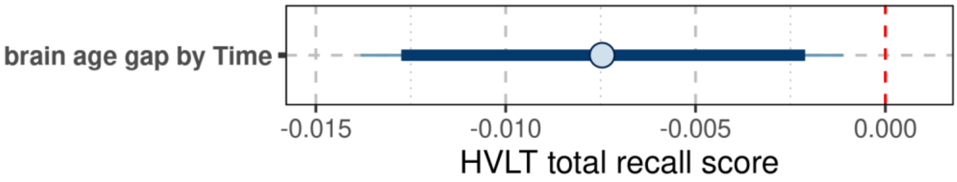

Mean (circle) and 90% (thick blue line) and 95% (thin blue line) credible intervals for the effect of predictors on HVLT total recall as estimated from the mixed effect regression model. The vertical red dashed line indicates zero. The lower panel zooms in on the effect of brain-age gap by time.

Supplementary Figure 8: Rate of MoCA decline by brain age gap

Supplementary Figure 8a: Trajectories of change

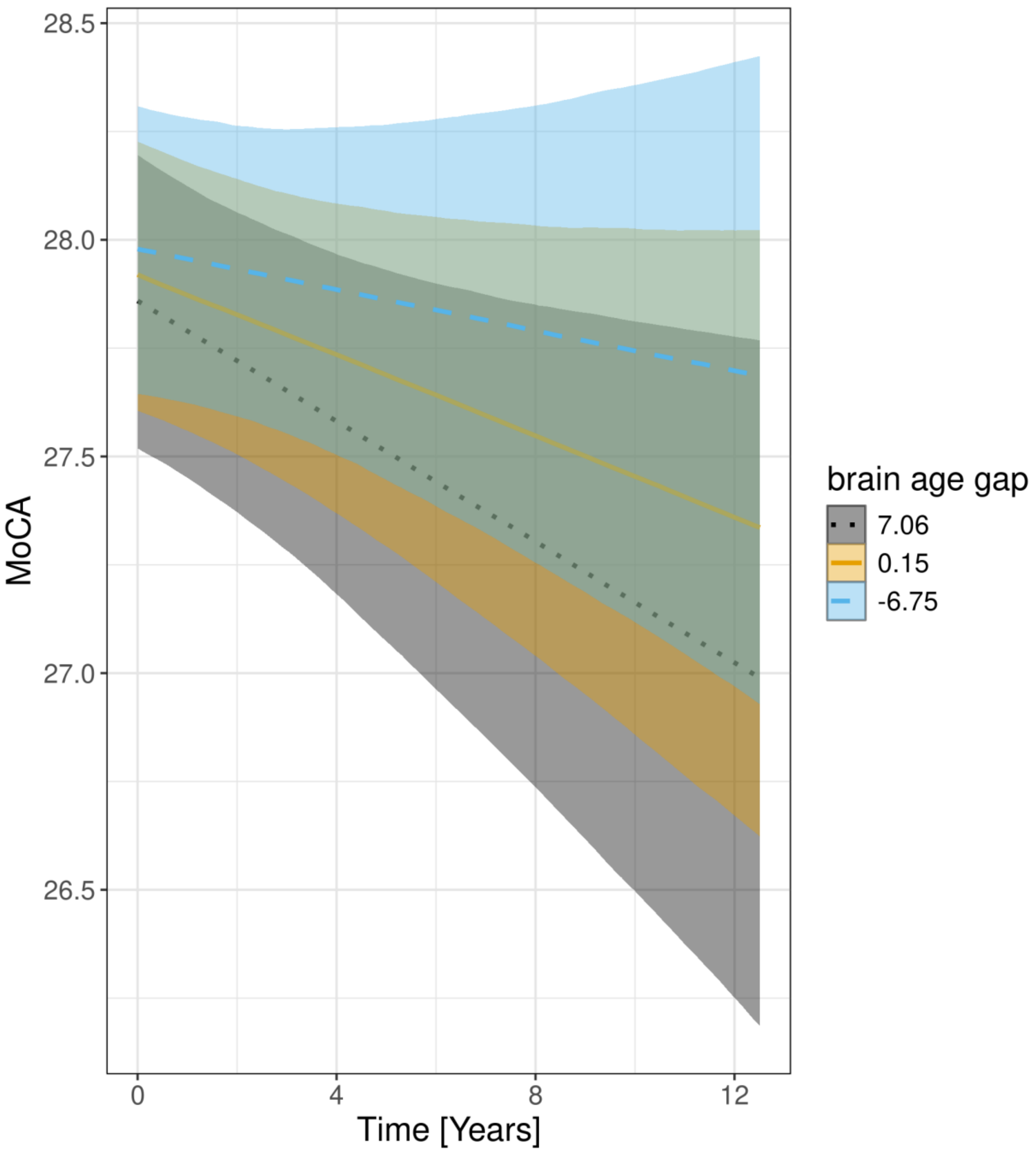

Marginal interaction effects of time with brain-age gap on Montreal Cognitive Assessment (MoCA) decline in generalized mixed effect models predicting MoCA by brain-age gap and diagnosis and their interaction with time, controlled for age, sex, education, and field strength with random slope and intercept terms for time, nested within individuals. There were 1,200 cases with 6,626 observations. Trajectories with 95% credible intervals are plotted for mean levels of the continuous variable brain-age gap and mean + 1 standard deviation and mean – 1 standard deviation.

Supplementary Figure 8b: Posterior distribution

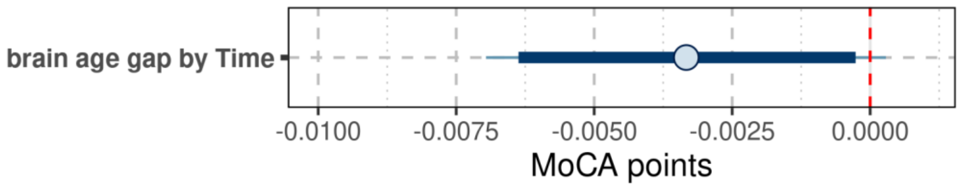

Mean (circle) and 90% (thick blue line) and 95% (thin blue line) credible interval for the effect of brain-age gap by time on the MoCA scores as estimated from the mixed effect regression model, controlling for diagnosis and its interaction with time, age, sex, education, and field strength. The vertical red dashed line indicates zero.

Supplementary Figure 9: Rate of UPDRS-3 score change by brain age gap

Supplementary Figure 9a: Trajectories of change

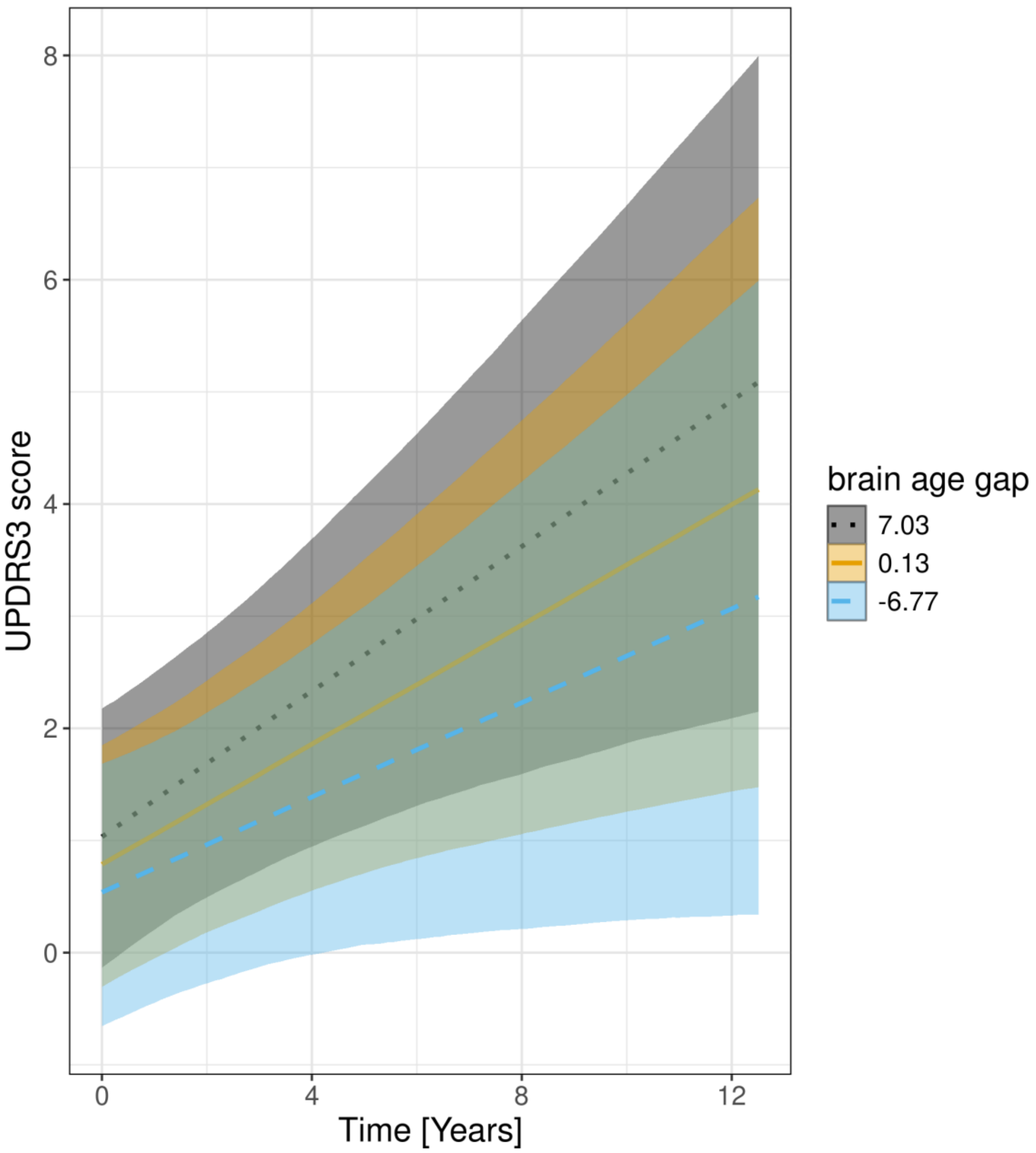

Marginal interaction effects of time with brain-age gap on Unified Parkinson's Disease Rating Scale-3 (UPDRS-3) score change in generalized mixed effect models predicting UPDRS-3 score by brain-age gap and diagnosis and their interaction with time, controlled for age, sex, education, and field strength with random slope and intercept terms for time, nested within individuals. There were 1,200 cases with 6,244 observations. Trajectories with 95% credible intervals are plotted for mean levels of the continuous variable brain-age gap and mean + 1 standard deviation and mean – 1 standard deviation.

Supplementary Figure 9b: Posterior distribution

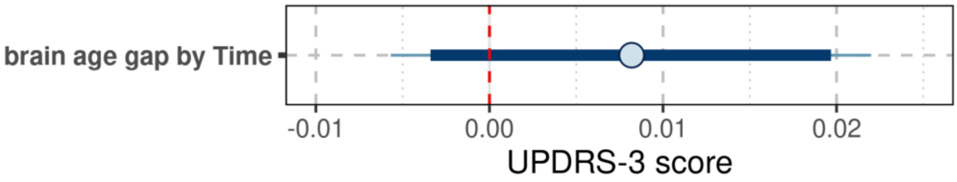

Mean (circle) and 90% (thick blue line) and 95% (thin blue line) credible interval for the effect of brain-age gap by time on the UPDRS-3 scores as estimated from the mixed effect regression model, controlling for diagnosis and its interaction with time, age, sex, education, and field strength. The vertical red dashed line indicates zero.

Supplementary Figure 10: MOCA score distribution

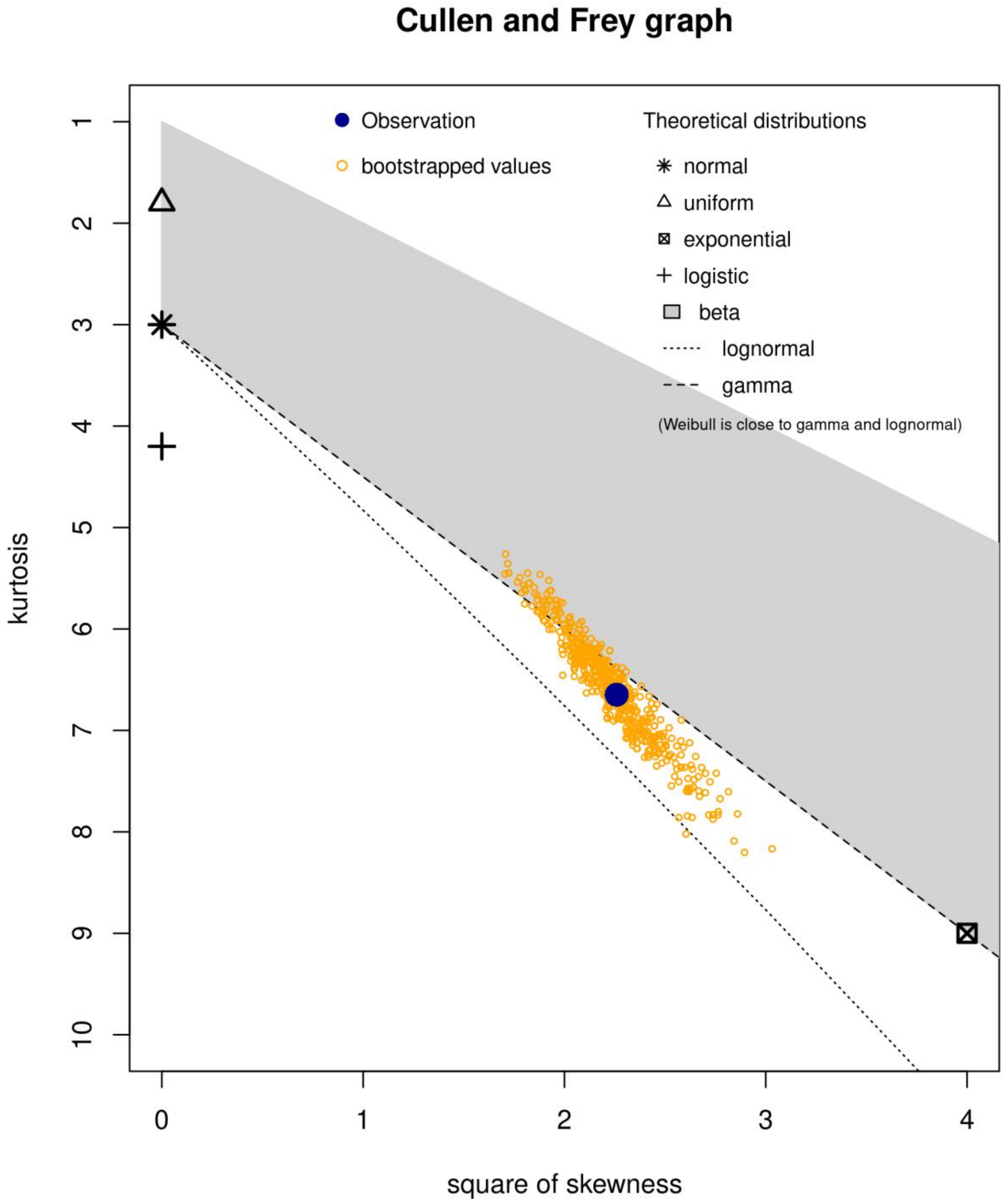

Cullen and Frey graph for the proximity of the Montreal Cognitive Assessment (MoCA) distribution to different theoretical distributions, including gamma, normal, beta-binomial, and log-normal distributions. The bootstrapped values of the MoCA distribution across 1,200 cases with 6,626 observations are plotted in orange, resembling most closely a gamma and a log-normal distribution.

Supplementary Figure 11: Histogram of MoCA residuals with different distributions

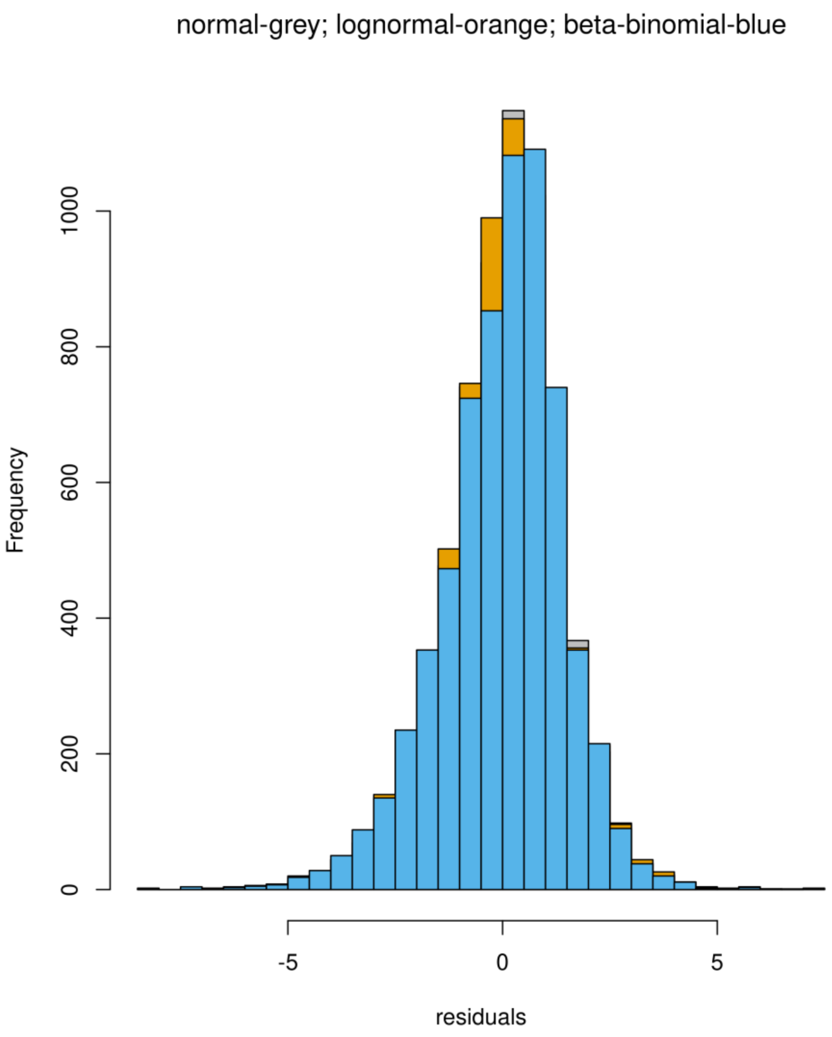

Histogram for the residuals of the generalized mixed effect models for Montreal Cognitive Assessment (MoCA) score as dependent variable, with brain-age gap and diagnosis and their interaction with time as predictors, controlled for age, sex, education, and field strength with random slope and intercept terms for time, nested within individuals. There were 1,200 cases with 6,626 observations. Histograms in grey plot the residual from the model using a Gaussian distribution, in blue for a beta-binomial distribution, and in orange for a log-normal distribution of the dependent variable. The residuals for a Gaussian distribution are at least as close to a normal distribution as the residuals of a log-normal or beta-binomial distribution, so that a Gaussian distribution was used for modelling the effect of time on longitudinal MoCA scores.

Supplementary References:

1. Beheshti I, Booth S, Ko JH. Differences in brain aging between sexes in Parkinson's disease. *NPJ Parkinsons Dis*. Feb 14 2024;10(1):35. doi:10.1038/s41531-024-00646-w
2. Eickhoff CR, Hoffstaedter F, Caspers J, et al. Advanced brain ageing in Parkinson's disease is related to disease duration and individual impairment. *Brain Commun*. 2021;3(3):fcab191. doi:10.1093/braincomms/fcab191
3. Beheshti I, Mishra S, Sone D, Khanna P, Matsuda H. T1-weighted MRI-driven Brain Age Estimation in Alzheimer's Disease and Parkinson's Disease. *Aging Dis*. May 2020;11(3):618-628. doi:10.14336/AD.2019.0617
4. Charisse D, Erus G, Pomponio R, et al. Brain age and Alzheimer's-like atrophy are domain-specific predictors of cognitive impairment in Parkinson's disease. *Neurobiology of aging*. Jan 2022;109:31-42. doi:10.1016/j.neurobiolaging.2021.08.020
5. Chen CL, Kuo MC, Wu WC, Hsu YC, Wu RM, Tseng WI. Advanced brain aging in multiple system atrophy compared to Parkinson's disease. *Neuroimage Clin*. 2022;34:102997. doi:10.1016/j.nicl.2022.102997
6. Chen CL, Hsu YC, Yang LY, et al. Generalization of diffusion magnetic resonance imaging-based brain age prediction model through transfer learning. *NeuroImage*. Aug 15 2020;217:116831. doi:10.1016/j.neuroimage.2020.116831
7. Kruschke JK. Bayesian Analysis Reporting Guidelines. *Nat Hum Behav*. Oct 2021;5(10):1282-1291. doi:10.1038/s41562-021-01177-7
8. Gelman A, Rubin DB. Inference from iterative simulation using multiple sequences (with discussion). *Stat Sci*. 1992;7(4):457–511.
