# Supplementary material for: Brain age in genetic and idiopathic Parkinson's disease
Source: Brain Commun. 2024 Dec 20;6(6):fcae382. doi: 10.1093/braincomms/fcae382 (PMC11660940; doi:10.1093/braincomms/fcae382)
Supplement: fcae382_Supplementary_Data [file fcae382_supplementary_data.zip › Supplementary-Materials-Example-Code.pdf]

Supplementary Materials: Example Code

```
#Load data

vorlage= read.csv("/.../Brainage_longformat.csv")  #load data in
long format (each row is one timepoint)

library(brms)
library("rstan")
options(mc.cores = parallel::detectCores())
rstan_options(auto_write = TRUE)

#example code for longitudinal model of cognitive score by brain
age and covariates; SDMT: Symbol Digits Modalities Test

b_sdmt_tr = brm(SDMT ~ diagnosis*Time + brainage_gap*Time + age +
sex + education + fieldStrength + (1 + Time|PATNO), data = vorlage,
warmup = 2000, iter = 8000, chains = 4, save_pars = save_pars(all =
TRUE), backend = "cmdstanr", threads = threading(4))

summary(b_sdmt_tr) # overview of model results

pp_check(b_sdmt_tr)  # posterior predictive check (determines how
samples from the posterior distribution fit the distribution of the
dependend variable)


res= data.frame(residuals(b_sdmt_tr)) # checks the distribution of
the model residuals
hist(res$Estimate, 50)


library(fitdistrplus)  #asses fit of dtaa and residuals to differtn
distributions
library(logspline)
x= subset(vorlage$SDMT_TOT, vorlage$SDMT_TOT> -10)


descdist(res$Estimate, discrete = FALSE, boot= 500)

descdist(res$Estimate, discrete = TRUE)

descdist(x, discrete = FALSE, boot= 500)

descdist(x, discrete = TRUE)


#-------------------------------------------------------------------
------------------------------------------------
#Plot results
```

```
library(ggplot2)

tiff(filename = ".../long_sdmt_brain_age_gap.tiff", width = 2000,
height = 2200, compression = "zip", res= 300)  # Plot of cognitive
score by brain age over time
c1= plot(conditional_effects(b_sdmt_tr, effects=
"Time:brainage_gap"), plot = FALSE)[[1]] +
c1= c1 + xlab("Time [Years]") + ylab("SDMT") +
scale_color_manual(values=c("red", "#E69F00", "#56B4E9"))
c1$labels$fill = "brain age gap" +
scale_color_manual(values=c("red", "#E69F00", "#56B4E9"))
c1$labels$colour = "brain age gap"+
scale_color_manual(values=c("red", "#E69F00", "#56B4E9"))
c1= c1 + theme(text=element_text(size= 15))+ theme(panel.background
= element_rect(fill = "white", colour = "grey50"))
c1= c1 + theme(panel.grid.major = element_line(colour = "grey90"))
c1= c1 + theme(panel.grid.minor = element_line(colour = "grey90"))
c1 = c1 +  theme(panel.border = element_rect(colour = "black", fill
= NA))
c1

dev.off()


library(tidyverse)


labels_h1= c("b_fieldStrength","b_sexmale", "b_YearsOfEducation",
"b_ageAtMRI", "b_brainage_gap","b_diagnosisPD_GBA",
"b_diagnosisPD_idiopathic", "b_diagnosisPD_LRRK2",
             "b_diagnosisProdromal_GBA",
"b_diagnosisProdromal_LRRK2", "b_Time", "b_diagnosisPD_GBA:Time",
             "b_diagnosisPD_idiopathic:Time",
"b_diagnosisPD_LRRK2:Time", "b_diagnosisProdromal_GBA:Time",
"b_diagnosisProdromal_LRRK2:Time",
             "b_Time:brainage_gap")


tiff(filename = "/.../long_sdmt_brain_age_gap_mcmc.tiff", width =
2600, height = 3200, compression = "zip", res = 300) #  MCMC (Markov
Chain Monte Carlo) plotting functions

c1= mcmc_plot(b_sdmt_tr, variable =labels_h1, prob = 0.90,
prob_outer= 0.95) + geom_vline(xintercept=0, color= "red", linetype=
2, lwd=0.5)+  scale_y_discrete(limits= labels_h1,labels=c("field
strength","sex", "years of education","age", "brain age gap", "PD-
GBA","PD", "PD-LRRK2", "prodrom. GBA",

"prodrom. LRRK2","Time", "PD-GBA by Time",

"PD by Time", "PD-LRRK2 by Time", "prodrom. GBA by Time", "prodrom
LRRK2 by Time",

"brain age gap by Time")) +
```

```
  theme(panel.background = element_rect(fill = "white", colour =
"grey50"))+
  theme(panel.grid.major = element_line(colour = "grey", linetype=
2))+
  theme(panel.grid.minor = element_line(colour = "grey", linetype=
3))+
  theme(panel.border = element_rect(colour = "black", fill = NA)) +
theme(text=element_text(size= 12))
c1
dev.off()


tiff(filename = "/.../long_sdmt_brain_age_gap_only_mcmc.tiff",
     width = 1600, height = 320, compression = "zip", res = 300)

c1= mcmc_plot(b_hvlt_tr, variable ="b_Time:brainage_gap", prob =
0.90, prob_outer= 0.95) + scale_x_continuous (limits = c(-0.015,
0.001)) + geom_vline(xintercept=0, color= "red", linetype= 2,
lwd=0.5)+  scale_y_discrete(limits=
"b_Time_years:corrected_brainage_gap",labels=c("brain age gap by
Time")) +
  theme(aspect.ratio = .15) +
  theme(panel.background = element_rect(fill = "white", colour =
"grey50"))+
  theme(panel.grid.major = element_line(colour = "grey", linetype=
2))+
  theme(panel.grid.minor = element_line(colour = "grey", linetype=
3))+
  theme(panel.border = element_rect(colour = "black", fill = NA)) +
theme(text=element_text(size= 12)) +
  xlab("SDMT")
c1

dev.off()
```
